# Synthesis, Physicochemical Characterization, and Antimicrobial Evaluation of Halogen-Substituted Non-Metal Pyridine Schiff Bases

**DOI:** 10.3390/molecules29194726

**Published:** 2024-10-06

**Authors:** Alexander Carreño, Rosaly Morales-Guevara, Marjorie Cepeda-Plaza, Dayán Páez-Hernández, Marcelo Preite, Rubén Polanco, Boris Barrera, Ignacio Fuentes, Pedro Marchant, Juan A. Fuentes

**Affiliations:** 1 Laboratory of Organometallic Synthesis, Center of Applied NanoSciences (CANS), Facultad de Ciencias Exactas, Universidad Andres Bello, República 330, Santiago 8370186, Chile; rmguevara1994@gmail.com (R.M.-G.); dayan.paez@unab.cl (D.P.-H.); 2 Departamento de Ciencias Químicas, Facultad de Ciencias Exactas, Universidad Andres Bello, Av. República 275, Santiago 8370146, Chile; marjorie.cepeda@unab.cl; 3 Departamento de Química de los Materiales, Facultad de Química y Biología, Universidad de Santiago de Chile, Av. Libertador B. O’Higgins 3363, Santiago 9170022, Chile; 4 Facultad de Ingeniería, Universidad Finis Terrae, Av. Pedro de Valdivia 1509, Santiago 7501015, Chile; 5 Departamento de Química Orgánica, Facultad de Química y de Farmacia, Pontificia Universidad Católica de Chile, Santiago 7820436, Chile; mpreite@uc.cl; 6 Laboratorio de Hongos Fitopatógenos, Centro de Biotecnología Vegetal (CBV), Facultad de Ciencias de la Vida, Universidad Andres Bello, República 330, Santiago 8370186, Chile; rpolanco@unab.cl; 7 Escuela de Tecnología Médica, Facultad de Salud, Universidad Santo Tomás, Santiago 8370003, Chile; bobarrera@hcuch.cl; 8 Laboratorio de Genética y Patogénesis Bacteriana, Facultad de Ciencias de la Vida, Universidad Andres Bello, República 330, Santiago 8370186, Chile; ignaciofuentesc547@gmail.com (I.F.); marchant573@gmail.com (P.M.); 9 Doctorado en Biotecnología, Facultad de Ciencias de la Vida, Universidad Andres Bello, República 330, Santiago 8370186, Chile

**Keywords:** pyridine Schiff bases, intramolecular hydrogen bond, antimicrobial activity, ROS

## Abstract

Four synthetic Schiff bases (PSB1 [(E)-2-(((4-aminopyridin-3-yl)imino)methyl)-4,6-dibromophenol], PSB2 [(E)-2-(((4-aminopyridin-3-yl)imino)methyl)-4,6-diiodophenol], PSB3 [(E)-2-(((4-aminopyridin-3-yl)imino)methyl)-4-iodophenol], and PSB4 [(E)-2-(((4-aminopyridin-3-yl)imino)methyl)-4-chloro-6-iodophenol]) were fully characterized. These compounds exhibit an intramolecular hydrogen bond between the hydroxyl group of the phenolic ring and the nitrogen of the azomethine group, contributing to their stability. Their antimicrobial activity was evaluated against various Gram-negative and Gram-positive bacteria, and it was found that the synthetic pyridine Schiff bases, as well as their precursors, showed no discernible antimicrobial effect on Gram-negative bacteria, including *Salmonella* Typhi (and mutant derivatives), *Salmonella* Typhimurium, *Escherichia coli*, and *Morganella morganii*. In contrast, a more pronounced biocidal effect against Gram-positive bacteria was found, including *Bacillus subtilis*, *Streptococcus agalactiae*, *Streptococcus pyogenes*, *Enterococcus faecalis*, *Staphylococcus aureus*, and *Staphylococcus haemolyticus*. Among the tested compounds, PSB1 and PSB2 were identified as the most effective against Gram-positive bacteria, with PSB2 showing the most potent biocidal effects. Although the presence of reactive oxygen species (ROS) was noted after treatment with PSB2, the primary mode of action for PSB2 does not appear to involve ROS generation. This conclusion is supported by the observation that antioxidant treatment with vitamin C only partially mitigated bacterial inhibition, indicating an alternative biocidal mechanism.

## 1. Introduction

Schiff bases represent a class of chemical compounds characterized by a distinctive functional group, azomethine (–N=C–), featuring a carbon–nitrogen double bond. This azomethine group serves as a central link within structures with the general formula (R1)–N=C–(R2)(R3), where any of the substituents, namely R1, R2, or R3, can be aryl or alkyl groups, among other possible substituents [1]. It is known that Schiff bases exhibit diverse applications, including their use as polymer stabilizers [2,3], intermediates in organic synthesis [4], catalysts [5], pigments [5], and dyes [6,7,8,9,10,11,12,13,14,15].

Due to their structural similarity to naturally occurring biological molecules, Schiff bases hold significant importance in biological contexts [16,17]. Metallic Schiff bases and non-metallic Schiff bases differ in chemical composition and properties. Metallic Schiff bases form coordination complexes with metal ions, whereas non-metallic Schiff bases lack metal ions in their structure [18]. The complexes formed by Schiff bases with metal ions have demonstrated enhanced efficacy compared to the ligands alone, suggesting that coordination with metals amplifies their antimicrobial properties [19,20,21,22,23]. Furthermore, Schiff base metal complexes have shown potent radical scavenging activities, sometimes exhibiting similar efficacy to standard antioxidants, such as ascorbic acid (vitamin C) [24]. On the other hand, the antimicrobial effect of non-metallic Schiff bases is primarily attributed to the azomethine group (–C=N–) present in their structure, which is available due to the lack of the metal ion. The presence of this group facilitates the formation of hydrogen bonds with the active sites of enzymes in microorganisms, which could inhibit enzyme activity and disrupt metabolic pathways or structures essential for microbial survival [18,25,26]. Additionally, the azomethine group can enhance the lipophilicity of the molecule, allowing it to penetrate microbial cell membranes more effectively, thereby increasing cell uptake [27]. Furthermore, the electronic properties of the azomethine group, such as its ability to delocalize electrons, contribute to the stability and reactivity of Schiff bases, enhancing their interaction with microbial cells. This electron delocalization can generate reactive oxygen species (ROS) within microbial cells, causing oxidative stress and damaging cellular components like DNA, proteins, and lipids [25,28]. While the azomethine group is a significant contributor to the antimicrobial activity, the overall efficacy of non-metal Schiff bases can also be influenced by other structural features, such as the presence of additional substituents that can modulate the electronic and steric properties of the molecule. These substituents can further enhance the antimicrobial effect by improving the molecule’s ability to interact with specific microbial targets or increasing its solubility and stability in biological environments [25]. Nevertheless, other structural features can also support the enhancement of the overall antimicrobial efficacy of these compounds.

Metallic and non-metallic Schiff bases differ significantly in their synthesis methods. The synthesis of the former involves the condensation of a metal ion with a Schiff base ligand formed through the reaction of an aldehyde or ketone with a primary amine [29,30]. During this process, the metal ion coordinates with the Schiff base ligand, forming a complex. This synthesis often requires metal salts and involves the removal of phenolic hydrogen to facilitate Schiff base formation [17,18]. Conversely, non-metallic Schiff bases are synthesized solely by condensing an aldehyde or ketone with a primary amine, thus forming the Schiff base ligand without metal ion coordination and simplifying the synthetic procedure [18,30].

Our research focuses on synthesizing and characterizing non-metal pyridine Schiff bases. These compounds consist of a pyridine ring and a phenolic ring connected by an azomethine group. An intramolecular hydrogen bond (IHB) in these Schiff bases contributes to their stability and antimicrobial properties [31,32,33]. These Schiff bases can also serve as ligands for d^6^-derived complexes, which have potential applications as fluorophores [34,35,36,37,38]. We are also interested in a better understanding of how substituent groups could enhance the potential antimicrobial properties of these compounds.

The pyridine ring in non-metal pyridine Schiff bases plays a crucial role in their antifungal and antibacterial activities. Studies have shown that the pyridine ring’s nitrogen atom significantly enhances Schiff bases’ antifungal activity. For example, compounds such as (*E*)-2-{[(3-aminopyridin-4-yl)imino]-methyl}-4,6-di-*tert*-butyl-phenol and (*E*)-2-{[(3-aminopyridin-4-yl)imino]-methyl}-4,6-di-chloro-phenol (L3), two examples of pyridine Schiff bases (PSBs), have shown antifungal effects against *Cryptococcus* spp. (fungus, yeast) [32,39]. In contrast, the compound (*E*)-2-[(2-aminophenyl)iminomethyl]-4,6-di-*tert*-butylphenol (L6), which lacks a pyridine ring, showed no significant antifungal activity [32]. These findings underscore the importance of the nitrogen atom in the pyridine ring for the antifungal efficacy of these Schiff bases, highlighting the structure–activity relationship.

Moreover, the phenolic moiety in non-metal pyridine Schiff bases also contributes to their biocidal activity. Halogen substitution on the phenolic ring has been found to enhance the antifungal activity. Specifically, the non-metal pyridine Schiff bases containing a phenol substituted with halogens, such as chlorine and fluorine, exhibit promising antifungal activity [31,32]. Additionally, the position of fluorine atoms on the phenolic ring influences receptor selectivity, suggesting that fluorine’s presence and position are crucial for designing effective antifungal compounds [31,32]. However, a systematic study evaluating the impact of other halogens on the phenolic ring of these Schiff bases has yet to be conducted on bacteria.

To date, the antimicrobial efficacy of non-metal pyridine Schiff bases has only been assessed against *Salmonella enterica*, a Gram-negative bacterium of the *Enterobacteriaceae* family [31,32]. The activity of these compounds against other bacteria, including Gram-positive bacteria (another kind of bacterial cell model), remains unexplored. Further research is necessary to understand the antimicrobial potential of non-metal pyridine Schiff bases across a broader spectrum of bacterial species.

**Figure 1:**
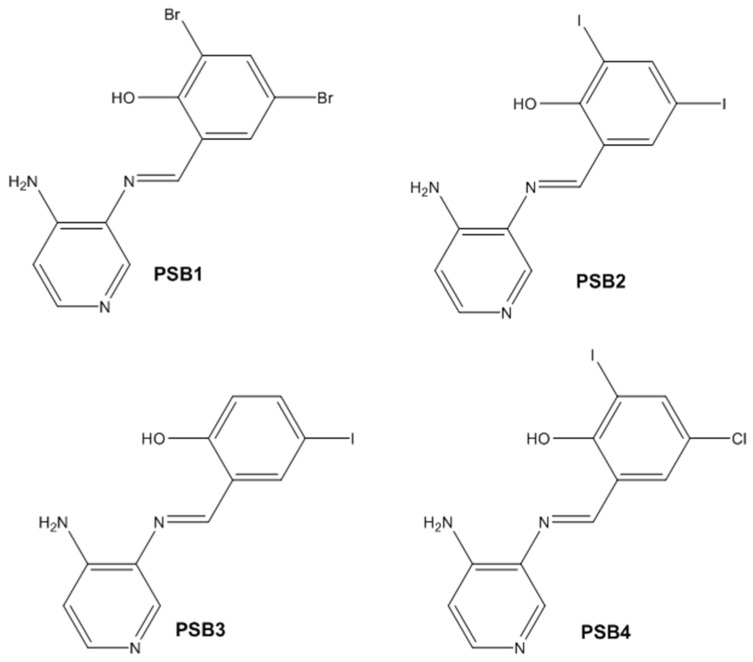
Chemical structures of (*E*)-2-(((4-aminopyridin-3-yl)imino)methyl)-4,6-dibromophenol (PSB1), (*E*)-2-(((4-aminopyridin-3-yl)imino)methyl)-4,6-diiodophenol (PSB2), (*E*)-2-(((4-aminopyridin-3-yl)imino)methyl)-4-iodophenol (PSB3), and (*E*)-2-(((4-aminopyridin-3-yl)imino)methyl)-4-chloro-6-iodophenol (PSB4).

In this study, we synthesized and characterized a new series of non-metal pyridine Schiff bases (PSBs) [(R1)–N=C–(R2)(R3), where R1 is a pyridine ring, R2 is hydrogen, and R3 is a substituted phenolic ring]. The phenolic ring is substituted with halogens (Br, I, and/or Cl) at the 4 and/or 6 positions, including a dibrominated substitution [PSB1, (*E*)-2-(((4-aminopyridin-3-yl)imino)methyl)-4,6-dibromophenol], a diiodized substitution [PSB2, (*E*)-2-(((4-aminopyridin-3-yl)imino)methyl)-4,6-diiodophenol], a monoiodide substitution [PSB3, (*E*)-2-(((4-aminopyridin-3-yl)imino)methyl)-4-iodophenol], and a monoiodide and monochlorinated substitution [PSB4, (E)-2-(((4-aminopyridin-3-yl)imino)methyl)-4-chloro-6-iodophenol] (Figure 1). These compounds were fully characterized using elemental analyses, FTIR, ^1^H and ^13^C-NMR, DEPT, D_2_O exchange, HHCOSY, TGA, MS, UV-Vis, quantum theoretical studies, and antimicrobial assays. All of these Schiff bases exhibit an intramolecular hydrogen bond between the –OH group of the phenolic ring and the nitrogen of the azomethine group. Additionally, we observed that the nature and position of the various halogens tested slightly influenced the electronic distribution of each molecule.

The antimicrobial activity study of these non-metallic pyridine Schiff bases revealed that while these compounds exhibited no significant effects against Gram-negative bacteria, they demonstrated pronounced biocidal effects against Gram-positive bacteria. Specifically, PSB1 and PSB2 were the most effective, with PSB2 showing the most potent biocidal effects. Despite the presence of reactive oxygen species (ROS) formed after the treatment with PSB2 in bacteria, the primary mode of action for PSB2 does not appear to involve ROS generation, as antioxidant treatment with vitamin C only partially mitigated bacterial inhibition, indicating an alternative biocidal mechanism. Thus, we found that the nature of the substitution of halogens in the phenolic ring of this kind of non-metal pyridine Schiff influences the biocidal activity of these compounds.

## 2. Results and Discussion

### 2.1. Synthesis and Characterizations

The corresponding phenol-substituted pyridine Schiff bases were prepared through the condensation of diaminepyridine and the corresponding substituent phenol aldehyde, following the previous procedure [18]. The chemical structure of the corresponding compounds (*E*)-2-(((4-aminopyridin-3-yl)imino)methyl)-4,6-dibromophenol (PSB1), (*E*)-2-(((4-aminopyridin-3-yl)imino)methyl)-4,6-diiodophenol (PSB2), (*E*)-2-(((4-aminopyridin-3-yl)imino)methyl)-4-iodophenol (PSB3), and (*E*)-2-(((4-aminopyridin-3-yl)imino)methyl)-4-chloro-6-iodophenol (PSB4) is shown in Figure 1. All the compounds were obtained with a high yield (more than 75%). Characteristic constants (molecular weight, yield, solid color) are shown in Table S1. On the other hand, all the PSBs tested demonstrated high solubility in DMSO, with PSB1 and PSB3 also showing solubility in methanol at room temperature. PSB1 and PSB2 exhibited low solubility in dichloromethane or acetonitrile, whereas PSB3 was soluble in both solvents at room temperature. PSB4 displayed low solubility in acetonitrile but was soluble in dichloromethane at room temperature.

Regarding decomposition points, PSBs melted at 211–212 °C, 189–190 °C, 163–164 °C, and 195–196 °C, respectively, before decomposition. Notably, PSB1 (di-halogen-substituted in the phenolic ring) exhibited higher melting points than PSB3 (which has one –I substituent in the phenolic ring). These differences can be attributed to the nature of the substituents, as illustrated in Figure 1. Additionally, the narrow interval corresponding to the decomposition points indicates the purity of the compounds.

Because PSB1 to PSB4 are newly synthesized compounds, we conducted an elemental (C, H, N) microanalysis, which confirmed the proposed formulas: C_12_H_9_Br_2_N_3_O for PSB1, C_12_H_9_I_2_N_3_O for PSB2, C_12_H_10_IN_3_O for PSB3, and C_12_H_9_ClIN_3_O for PSB4. The mass spectrometry results further supported these molecular formulas, with observed peaks at 371.9, 465.7, 339.9, and 373.8 for PSBs, respectively (Figures S1–S4).

**Figure 2:**
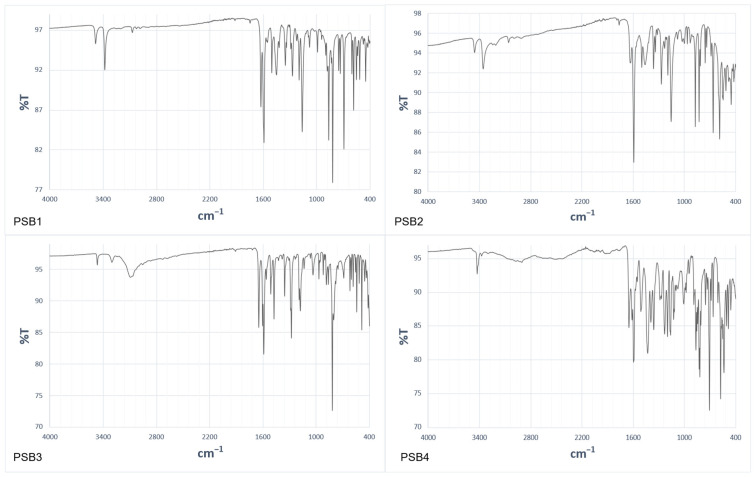
FTIR (ATR) spectrum of PSB1, PSB2, PSB3, and PSB4.

For a comprehensive physicochemical characterization, we employed thermogravimetric analysis (TGA) (Figures S5–S8) to compare the thermal decomposition temperatures. This analysis revealed a weight loss at approximately 211 °C for PSB1, 189 °C for PSB2, 163 °C for PSB3, and 195 °C for PSB4, consistent with their respective decomposition points reported above. Additionally, we obtained ATR-FTIR spectra showing several bands between 2000 and 4000 cm^−1^ (Figures S9–S12). Due to the limitations of ATR [33], the –OH bands appeared thinner than those obtained with KBr pellets. Figure 2 shows the FTIR spectrum for PSB1 to PSB4. The main absorption frequencies showed asymmetric and symmetric bands corresponding to νNH_2_ vibrations (3483 and 3380 cm^−1^ for PSB1, 3454 and 3352 cm^−1^ for PSB2, 3465 and 3298 cm^−1^ for PSB3, 3425 and 3372 cm^−1^ for PSB4) and the νOH band around 3067 cm^−1^, indicating the presence of intramolecular hydrogen bonding (IHB). Other studies have reported broader bands in the 3500–3000 cm^−1^ range due to νOH stretching involved in IHB [34,35]. However, in our FTIR spectra, these bands likely overlapped with the νNH_2_ bands. For all compounds, the absorptions around 1626–1648 cm^−1^ were assigned to –C=N– (azomethine group) stretching, and the absorptions around 1591 cm^−1^ corresponded to –C=C– stretching, as previously reported for similar compounds [31,32,36]. The computed frequencies (see below) agreed with the experimental data (Table S2, Figures S13–S16).

**Figure 3:**
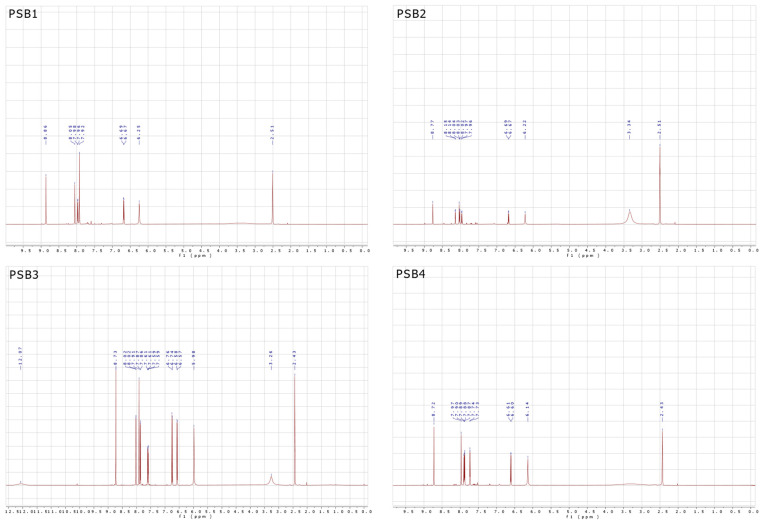
^1^H-NMR spectrum of the corresponding PSB1 to PSB4 at 400 MHz and 25 °C, with samples dissolved in deuterated DMSO.

NMR studies were conducted to confirm the structures of all PSBs. The ^1^H-NMR spectrum in DMSO-d_6_ as the solvent (Figure 3, see Figure S17 for arbitrary proton numbering) showed a broad peak at 12.07 ppm, which was assigned to the –OH group in PSB3, indicating intramolecular hydrogen bonding (IHB). This peak was not observed for PSB1, PSB2, or PSB4, which could be attributed to the presence of electron-donating groups, leading to out-of-range values in the NMR spectrum [32] (Table S3, with ^1^H-NMR spectra provided in Figures S18–S21). The behavior of the –OH group’s peak is consistent with observations of similar compounds, which depend on the nature and number of substituents in the phenolic ring [31,32,36,37].

**Figure 4:**
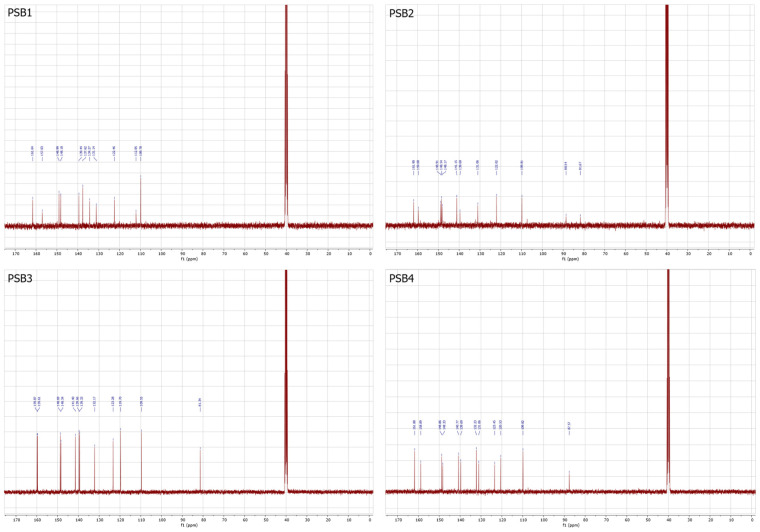
^13^C-NMR spectrum of the corresponding PSB1 to PSB4 at 100 MHz and 25 °C, with samples dissolved in deuterated DMSO.

The amino proton (–NH_2_) appeared at 6.25 ppm for PSB1, 6.22 ppm for PSB2, 5.98 ppm for PSB3, and 6.14 ppm for PSB4. The –CH=N– azomethine proton (H4) appeared as a singlet at 8.86 ppm, 8.77 ppm, 8.73 ppm, and 8.72 ppm for PSB1, PSB2, PSB3, and PSB4, respectively, as reported for similar compounds [36,37,38,39,40]. The D_2_O exchange confirmed the assignment of the amino group’s protons (–NH_2_) for PSB1 to PSB4 and the –OH proton for PSB3 (Figures S22–S25). The three protons in the pyridine ring (H1 to H3) appeared between 7.97 and 7.85 ppm, 6.68 and 6.57 ppm, and 8.06 and 7.91 ppm, respectively. The protons in the phenolic ring appeared at 7.92 ppm (H5 and H6, signal overlapping) for PSB1, 8.15 ppm (H5) and 8.06–8.00 ppm (H6) for PSB2, 8.02 ppm (H5), 7.60 ppm (H6), and 6.75 ppm (H7) for PSB3, and 7.93–7.85 ppm (H5) and 7.73 ppm (H6) for PSB4. In PSB3, the protons shifted to lower fields than PSB1, PSB2, and PSB4 due to increased nuclear magnetic shielding. For PSB3, the H7 proton was observed, highlighting the difference between mono-halogen and di-halogen substituted PSBs in the phenolic ring. HHCOSY confirmed all of these assignments (Figures S26–S29). To further advance the structural characterization, the ^13^C-NMR spectra of PSBs were obtained (Figure 4; see Figure S30 for the arbitrary numbering of carbon atoms and Figures S31–S34 for the ^13^C-NMR spectra). In this analysis, a signal typically attributed to the azomethine carbon was observed at 161.64 ppm, 161.90 ppm, 159.87 ppm, and 161.88 ppm for PSB1, PSB2, PSB3, and PSB4, respectively, which is consistent with similar compounds [41,42]. Additionally, the signal corresponding to the C12 carbon bearing the –OH substituent was found at 157.03 ppm for PSB1, 159.68 ppm for PSB2, 159.61 ppm for PSB3, and 158.89 ppm for PSB4. To further differentiate quaternary carbons from tertiary ones, a DEPT 45 analysis was performed, which confirmed the carbon skeletons of all compounds tested (Figures S35–S38). These analyses collectively confirm the proposed structures and the purity of all pyridine Schiff bases.

### 2.2. UV-Vis Studies

**Figure 5:**
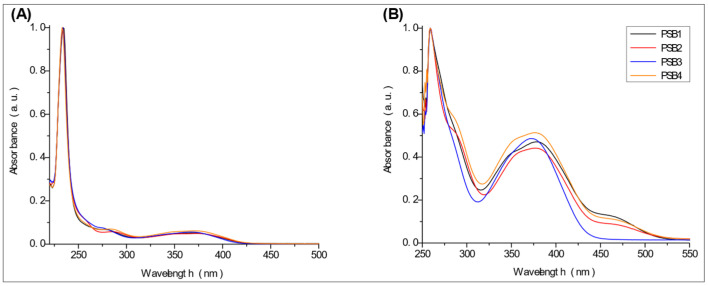
UV-VIS absorption spectra of PSBs under study in dichloromethane (**A**) or DMSO (**B**) at room temperature.

The absorption spectra of the compounds were recorded in aerated solutions in dichloromethane (ε = 8.93) and DMSO (ε = 46.7) at room temperature, utilizing organic solvents of varying polarity [43,44]. The spectra revealed two prominent absorption bands centered at 234 nm and 370 nm in dichloromethane and 259 nm and 372–380 nm in DMSO. These bands can be assigned predominantly to n→π*/π→π* and π→π* transitions, respectively, which is consistent with observations of similar compounds [36,38,45,46] (Figure 5 and Table S4).

The absorption band at 234 nm or 259 nm was assigned to the HOMO → LUMO+4 transition for PSB1, the HOMO-8 → LUMO transition for PSB2, the HOMO-6 → LUMO transition for PSB3, and the HOMO-2 → LUMO+2 transition for PSB4 (see below). The band located at 370–380 nm was attributed to the HOMO → LUMO transition (see Table S5). For all PSBs, the electron density in the HOMO is distributed across the entire molecule, with greater localization on the pyridine ring in PSB1and PSB4 and the phenolic ring in PSB2 and PSB3, reflecting the influence of halogen electronegativity. The LUMO orbital is primarily localized on the phenolic ring and, to a greater extent, the azomethine group within the six-membered ring formed by the intramolecular hydrogen bond (IHB) in both molecules (for molecular orbitals involved in the electronic transitions, see Table S6). The electronic absorption data in dichloromethane and DMSO are summarized in Table S4. In all cases, computed calculations are consistent with the experimental data (see Tables S5 and S6). This will be further discussed in Section 2.3 (Theoretical Analysis).

Overall, both absorption bands depend on solvent polarity, exposing a blue shift of the maximum absorption wavelength as the solvent polarity increases. The position of the two absorption bands is not significantly affected by the presence of mono-halogenated or di-halogenated substituents on the phenolic ring, a pattern observed in both solvents tested (Figure 5). The results with DMSO indicate a slight red shift for PBS3 compared to PBS1, PBS2, and PBS4 in the polar aprotic solvent (DMSO). In contrast, no significant shift (approximately 2 nm) in the absorption band corresponding to the π→π* transition was observed when an apolar organic solvent, such as dichloromethane, was used. These results suggest the stability of the IHB in these compounds. Similar PSBs with an IHB have been reported to form a six-membered chelate ring between the –OH group and the nitrogen atom of the azomethine group, conferring high stability [47].

Figure S39 displays the emission spectra of PBSs in dichloromethane. All compounds exhibit very low emission intensity, as corroborated by their quantum yield values (Table S4). Previous studies on the luminescence properties of similar systems containing the azomethine group have concluded that emission results from structural rigidity, significantly reducing vibronic relaxation [48,49,50]. For all PSBs, the observed low luminescence aligns with the molecular configuration changes upon excitation.

As previously discussed, the critical transitions in these molecules involve the LUMO orbital, which has an antibonding character in the azomethine group region. This characteristic implies that the excited state does not maintain the double-bond character between the aromatic rings due to the population of the LUMO orbital, resulting in the loss of the coplanar configuration. Consequently, the excited state loses its rigidity, decreasing the emission probability. This behavior is consistent with studies on photoisomerization, where conformational changes follow optical excitation in the C=C or N=N double bonds [51,52,53,54].

### 2.3. Theoretical Analysis

The optimized parameters for the PSBs are detailed in Table S7. The C-O bond distances for PSB1, PSB2, and PSB4 were observed at approximately 1.337 Å, while PSB3 exhibited a slightly longer distance at 1.344 Å. The O-H bond distances displayed a similar trend. Regarding the azomethine group (C=N), bond lengths were consistently around 1.285 Å for all PSBs, aligning with values reported for analogous compounds [18,31,36]. The bond lengths for PSB1, PSB2, and PSB4 were approximately 1.738 Å, whereas PSB3 showed a longer distance of 1.785 Å, reflecting the influence of mono-halogen versus di-halogen substitution in the phenolic ring (see atom numbering in Figure S40).

Frequency calculations revealed that the asymmetric and symmetric modes of the –NH_2_ group appeared at 3712 cm^−1^ and 3646 cm^−1^ for all PSBs. The νOH, νC-H(azomethine), and νC=N(azomethine) modes were observed at 3186 cm^−1^, 3061 cm^−1^, and 1671 cm^−1^. PSB3 exhibited slight frequency shifts compared to the other PSBs, reflecting again the differences between mono- and di-halogen substitution in the phenolic ring. These calculated frequencies are summarized in Table S2 and are consistent with previously discussed experimental results.

#### 2.3.1. NBO Analysis

The NBO (natural bond orbital) analysis determined that the π-electrons in both aromatic rings are delocalized in all PSBs. Interactions involving the –NH_2_ and –OH substituents also stabilize the compounds. The detailed NBO results are presented in Table 1, and the atom numbering for the NBO analysis is provided in Figure S40.

#### 2.3.2. Optical Properties

To investigate UV-VIS transitions, we performed time-dependent density functional theory (TDDFT) calculations using dimethyl sulfoxide (DMSO) and dichloromethane (DCM) as solvents. Critical parameters, such as the most significant absorption bands, oscillator strengths, and corresponding transitions in DMSO, are summarized in Tables S5 and S6.

The calculated results align well with the experimental data, maintaining consistent trends across dichloromethane and DMSO solvents. To further support the assignments in Table S5, the isosurface plots of the relevant molecular orbitals are provided in Table S6. Concerning the absorption spectra, all molecules exhibit a first band between 220 and 244 nm, which is attributed to a π→π* transition for PSB1 and a combination of π→π*/n→π* transitions for PSB2 through PSB4 (Table S5). The second absorption band observed between 378 and 389 nm corresponds to a π→π* transition for all PSBs (assigned as HOMO → LUMO). In all cases, the electron density in the HOMO, which defines the energy of the highest occupied molecular orbital, is delocalized across the entire molecule, with the –I substituent in position 4 contributing most significantly among the halogen substituents (refer to Table S6). See Table S6 for molecular orbitals involved in electronic transitions. In contrast, the LUMO (lowest unoccupied molecular orbital) is primarily localized on the phenolic ring and, to a greater extent, on the azomethine group in all molecules. The azomethine group plays a crucial role in these electronic transitions (Table 1).

### 2.4. Antimicrobial Activity of Pyridine Schiff Bases

The study of non-metallic pyridine Schiff bases is essential due to their potential antimicrobial properties [31,32,36]. The pyridine ring, particularly its nitrogen atom, is critical for enhancing these compounds’ antifungal and antibacterial activities [32,36]. The halogenated phenolic ring also further amplifies their biocidal effects [31,32]. In this section, we aimed to evaluate the impact of halogen substitutions on the phenolic moiety by testing these Schiff bases against a wider variety of bacterial species. Expanding the scope of bacterial targets is essential for advancing the development of more effective antimicrobial agents, particularly in light of the growing challenge of antibiotic resistance.

To systematically evaluate the antimicrobial activity of the synthesized Schiff bases, we tested a range of bacteria representing diverse groups of microorganisms. Gram-negative bacteria, characterized by a distinct cell envelope structure comprising an inner cytoplasmic membrane and an outer membrane separated by the periplasmic space, were tested. The outer membrane of Gram-negative bacteria is notable for its composition of lipopolysaccharides (LPS) and porin proteins, which contribute to its permeability barrier and environmental resilience. Additionally, Gram-negative bacteria possess a relatively thin peptidoglycan layer in their cell wall [55]. These microorganisms are ubiquitous across various environments and encompass both pathogenic and non-pathogenic species, making them essential in microbiology, biotechnology, and medicine [55]. In this study, we evaluated the antimicrobial efficacy against *Salmonella* Typhi (a causative agent of typhoid fever [56]), *Salmonella* Typhimurium (which causes gastroenteritis [57]), *Escherichia coli* (associated with traveler’s diarrhea and urinary infections [58]), and *Morganella morganii* (an opportunistic pathogen [59]). Nevertheless, the pyridine Schiff bases and their synthetic precursors exhibited no discernible effect on the tested Gram-negative bacteria.

Since the lack of an antimicrobial effect of the pyridine Schiff bases on Gram-negative bacteria may be attributed to the outer membrane acting as a barrier, mutants with increased permeability were employed. Specifically, *Salmonella* Typhi mutants lacking the outer membrane protein OmpA (which contributes to membrane stability and barrier function) were utilized. Deletion of OmpA (*S.* Typhi Δ*ompA*) results in compromised outer membrane integrity, rendering the bacteria more susceptible to damage and facilitating antibiotic penetration [60]. The *yibP* mutant (*S.* Typhi Δ*yibP*) involved in bacterial envelope architecture was also tested. Deletion of *yibP* similarly impacts membrane permeability [60]. These mutants exhibit increased susceptibility to vancomycin, an antibiotic known for its inability to penetrate Gram-negative cells. This demonstrates that these mutations effectively enhance bacterial permeability, as previously shown for these and other genes related to the envelope integrity in *Salmonella enterica* [60,61].

None of the tested mutants with increased envelope permeability exhibited enhanced susceptibility to the pyridine Schiff bases tested. The pyridine Schiff bases exhibited no biocidal effect on Gram-negative bacteria, regardless of the presence of mutations impairing membrane integrity, such as the ones mentioned above. This lack of increased susceptibility may be explained by the absence of a target for these Schiff bases in Gram-negative bacteria or the inability of the Schiff bases to reach their potential target sites. Consequently, the mutations did not facilitate the observation of any antimicrobial activity, suggesting that the mode of action of these Schiff bases, if any, does not involve a mechanism present or accessible in Gram-negative bacteria.

RpoS plays a role in the antimicrobial resistance mechanisms of *Salmonella enterica*. Studies have demonstrated that RpoS is integral to regulating stress responses, influencing resistance to various challenges, including oxidative and thermal stresses, as well as antimicrobial agents [62,63,64]. Consequently, we tested *Salmonella* Typhi Δ*rpoS*, which exhibits diminished resistance to various stressors, particularly oxidative stress [63,64]. Nevertheless, pyridine Schiff bases exerted no antimicrobial effects on this mutant under the tested conditions. The lack of increased susceptibility in the *Salmonella* Typhi Δ*rpoS* mutant to the pyridine Schiff bases reinforces the previous findings with the *ompA* and *yibP* mutants. Despite the compromised resistance to oxidative stress and the increased permeability observed in these mutants, the pyridine Schiff bases did not exhibit any antimicrobial effects. This further supports the notion that the target for these Schiff bases, if present, either does not exist in Gram-negative bacteria or remains inaccessible to the compounds tested.

According to these results, which demonstrated no antimicrobial effects of the pyridine Schiff bases on the tested Gram-negative bacteria, we decided to evaluate the effect of these Schiff bases on Gram-positive bacteria. Gram-positive bacteria have a different cell envelope structure lacking the outer membrane that serves as a barrier in Gram-negative bacteria, potentially allowing for greater accessibility of the Schiff bases to their target sites [65,66,67]. Thus, we also tested Gram-positive bacteria, prokaryotic microorganisms characterized by the presence of a cytoplasmic membrane and a thicker cell wall compared to Gram-negative bacteria, to determine whether our compounds inhibited their growth. In this study, we evaluated *Bacillus subtilis* (sporulated bacteria producing multiple potentially beneficial metabolites [68]), *Streptococcus agalactiae* (neonatal meningitis [69]), *Streptococcus pyogenes* (impetigo, erysipelas, cellulitis, pharyngitis [70]), *Enterococcus faecalis* (bloodstream infections, infective endocarditis [71]), *Staphylococcus aureus* (a major human pathogen causing a wide range of clinical infections [72]), and *Staphylococcus haemolyticus* (nosocomial infections [73]).

In Gram-positive bacteria, we observed an inhibitory effect exerted by the pyridine Schiff bases. Specifically, PSB1 and PSB2 demonstrated more pronounced effects (Table 2). To estimate the contribution of the substituted phenolic and pyridine moieties of the corresponding pyridine Schiff bases to the antimicrobial activity, we also tested the corresponding phenolic aldehyde (precursor 1) and 3,4-diaminopyridine (common precursor 2 for all pyridine Schiff bases), following a previously reported strategy [18]. As shown in Table 2, precursor 2 exhibited no or a lower biocidal effect than the corresponding pyridine Schiff base. In contrast, the different precursors 1 showed distinct effects, with 3,5-dibromosalicyaldehyde (precursor for PSB1) and 3,5-diiodosalicyaldehyde (precursor for PSB2) exerting the most noticeable effects. Interestingly, the 3,5-diiodosalicyaldehyde precursor 1 exhibited the highest biocidal effect in all cases tested (Table 2).

PSB1 and PSB2 (Figure 1) showed the lowest MIC in the tested Gram-positive bacteria, indicating that these pyridine Schiff bases are the most effective biocidal compounds. PSB1 exhibited a similar effect to its corresponding precursor 1 (3,5-dibromosalicyaldehyde), suggesting that the phenolic moiety contributes the most to the antimicrobial effect in this case (Table 2). In contrast, PSB2 showed a better effect than its corresponding precursor 1 (3,5-diiodosalicyaldehyde), indicating that the interaction with precursor 2 (3,4-diaminopyridine, which exerts no effect by itself, through the azomethine link generates a synergistic antimicrobial effect (Table 2). Regarding PSB3 and PSB4, the latter tended to show a better biocidal effect, although neither was as effective as PSB2. Thus, pyridine Schiff bases PSB1 and PSB2 exhibited significant antimicrobial activity against Gram-positive bacteria, with PSB2 showing the most potent effect. The phenolic moiety was crucial for PSB1’s efficacy, while a synergistic interaction enhanced PSB2’s biocidal properties, highlighting their potential as effective antimicrobial agents.

It has been described that exposure to biocidal products, including antibiotics or other antimicrobial compounds, produces the so-called secondary reactive oxygen species (ROS) [74,75,76,77]. These secondary ROS (usually peroxide) can be produced due to the increased microbial aerobic metabolism induced by cell damage in the presence of the antimicrobial compound [74,78]. In this context, the ROS can also function as signaling molecules that activate stress responses within the bacterial cells (e.g., ROS-related regulons in bacteria, such as OxyR), indicating a dual role in the mechanism of action of biocidal products [74,76,77]. The production of secondary ROS occurs as a secondary effect of the antibacterial action of these products, contributing to oxidative stress within the bacterial cells and acting as an additional damaging agent that contributes to cell death by causing oxidative damage to cellular components [74,76,77]. Secondary ROS highlights the complex interplay between oxidative stress, cell damage, and stress response activation induced by exposure to biocidal products.

**Figure 6:**
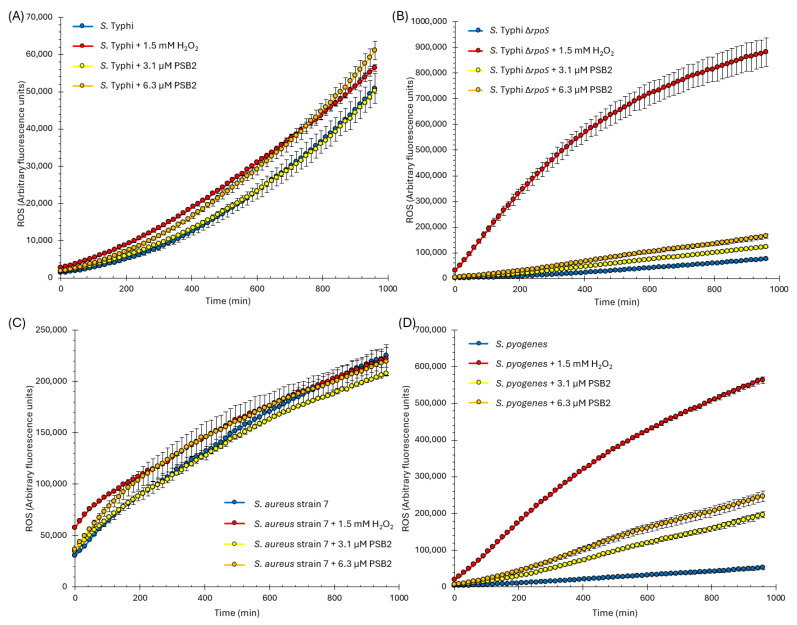
The intracellular ROS (reactive oxygen species) production was assessed in Gram-negative (**A**,**B**) and Gram-positive (**C**,**D**) bacteria following treatment with two concentrations of PSB2 (3.1 and 6.3 µM). For comparison, 1.5 mM of H_2_O_2_ was used as a positive control. We obtained similar results to those shown in (**C**) with *Staphylococcus aureus* strains 2 and 7 and *Staphylococcus haemolyticus*. Conversely, similar outcomes were observed with *Streptococcus agalactiae* and *Enterococcus faecalis*, as shown in (**D**) (Figure 7). With *Bacillus subtilis*, we observed intermediate results compared to (**C**,**D**). The bars represent the standard error of the mean (SEM) for n = 3. ROS was determined using the H_2_DCFDA probe.

To determine whether PSB2, the Schiff base with the most potent antimicrobial effect, induces the production of reactive oxygen species (ROS), we quantified the total intracellular ROS production using the H_2_DCFDA probe. The H_2_DCFDA probe is a chemical compound that detects reactive oxygen species (ROS) within cells, including bacteria, by emitting fluorescence when it reacts with ROS. This makes it helpful in studying oxidative stress and related cellular processes. The H_2_DCFDA probe enters bacteria and is cleaved by intracellular esterases. Upon oxidation by reactive oxygen species (ROS), it fluoresces, enabling ROS detection in live cells [79,80]. In this sense, we measured the intracellular ROS generated in Gram-negative bacteria following exposure to PSB2. It is important to note that PSB2 did not exhibit any antimicrobial effects on these bacteria. Figure 6A shows the analysis performed in *Salmonella* Typhi, but we obtained similar results with other Gram-negative bacteria tested (i.e., no effect). We observed that a concentration of 3.1 µM of PSB2 did not affect ROS generation beyond the levels produced by the normal metabolism of the bacteria (as indicated by comparing the blue and yellow curves). In contrast, the presence of 6.3 µM of PSB2 led to a slight increase in intracellular ROS accumulation comparable to the effect observed with 1.5 mM H_2_O_2_ (positive control, compare red and orange curves).

The observed slight intracellular ROS accumulation induced by PSB2 (Figure 6A) suggests that the compound can elicit some adverse effects in Gram-negative bacteria, evidenced by the generation of secondary intracellular ROS. However, the fact that PSB2 does not impact the minimum inhibitory concentration (MIC) of Gram-negative bacteria suggests that these organisms possess mechanisms to neutralize the effects of PSB2 and the secondary ROS it induces. This is further supported by their known repertoire of multiple ROS-resistance mechanisms [81,82]. Consequently, despite promoting a slightly concentration-dependent ROS accumulation, PSB2 fails to exert its antimicrobial effect. The bacteria’s robust ROS defense mechanisms likely mitigate the detrimental effects of PSB2, thus hindering its ability to effectively inhibit bacterial growth or survival.

*Salmonella enterica* has developed multiple strategies to counteract reactive oxygen species (ROS). One of its primary defenses involves ROS-scavenging enzymes, including superoxide dismutases, catalases, and peroxidases. These enzymes detoxify ROS, thus mitigating oxidative stress [82]. The inactivation of these enzymes results in heightened sensitivity to ROS and reduced survival within host cells [83]. On the other hand, the sigma factor RpoS (σS), considered a master stress response regulator, plays a significant role in the resistance of *Salmonella enterica* to oxidative stress. RpoS is crucial for bacterial survival under oxidative and other stresses [62]. RpoS remodels global gene expression, including the expression of ROS-detoxifying enzymes, to ensure the survival of quiescent cells under non-optimal growth conditions, including oxidative stress, by regulating a wide array of genes and physiological responses. In this context, we used an *rpoS*-deficient mutant of *Salmonella* Typhi (*S.* Typhi Δ*rpoS* [63,64]), which is more susceptible to oxidative and other types of stress due to lower expression of ROS detoxifying enzymes [84]. This allowed us to observe whether there would be an increased accumulation of ROS in the absence of RpoS. As expected, intracellular ROS increased under all tested conditions. However, the increase induced by PSB2 was less than that observed with the H_2_O_2_ control. This result aligns with the observations that the Δ*rpoS* mutant did not show changes in the MIC due to PSB2′s presence. Considering that a mutant with impaired ROS defense systems (such as the Δ*rpoS* mutant) exhibits only a modest increase in ROS in the presence of PSB2 and that PSB2 does not affect the MIC of this mutant, we infer that ROS generation would not be the primary mechanism of cellular damage caused by PSB2.

As the next step, we aimed to compare the ROS accumulation in *Staphylococcus* spp. (a bacterium with robust oxidative stress defense systems, notably including catalase) with *Streptococcus* spp., a species more susceptible to oxidative stress due to the absence of the catalase enzyme [85,86]. This comparison would help us understand the differential impact of PSB2 on bacteria with varying oxidative stress defense mechanisms. To conduct this comparison, we tested *Staphylococcus aureus* strain 7 (though similar results were obtained with other *Staphylococcus* strains tested in Table 2) and compared it with *Streptococcus pyogenes* (with similar results observed for other *Streptococcus* strains in Table 2). As expected, ROS accumulation in *Staphylococcus aureus* strain 7 remained relatively constant regardless of adding H_2_O_2_ or PSB2 (Figure 6), aligning with robust anti-ROS mechanisms, including catalase. In contrast, a significant increase in ROS accumulation was noted in *Streptococcus pyogenes* in the presence of H_2_O_2_, and a concentration-dependent ROS accumulation was also observed with PSB2, consistent with the lower oxidative stress tolerance of this strain.

However, both *Staphylococcus aureus* and *Streptococcus pyogenes* demonstrated susceptibility to PSB2 (Table 2), indicating that the primary biocidal mechanism of PSB2 is not ROS generation. The ROS observed appears to be secondary, reinforcing the conclusions obtained with Gram-negative bacteria. Therefore, PSB2 likely exerts its bactericidal effect through other pathways, with ROS accumulation resulting from cellular damage rather than the primary cause (Figure 6).

**Figure 7:**
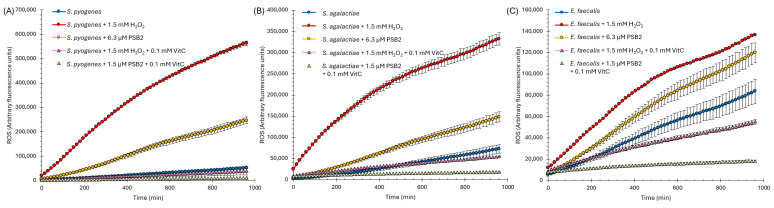
Effect of vitamin C (VitC) on quenching ROS (reactive oxygen species) action in *Streptococcus pyogenes* (**A**), *Streptococcus agalactiae* (**B**), or *Enterococcus faecalis* (**C**). Bacteria were treated with PSB2 (6.3 µM). For comparison, 1.5 mM of H_2_O_2_ was used as a positive control. The bars represent the standard error of the mean (SEM) for n = 3. ROS was determined using the H_2_DCFDA probe.

To understand the contribution of ROS to the biocidal effect exerted by PSB2, we first aimed to verify whether we could neutralize or diminish the impact of ROS. For this purpose, we employed vitamin C. Vitamin C, known for its potent antioxidant properties, can reduce oxidative stress by neutralizing free radicals and decreasing reactive oxygen species (ROS) levels [86]. We selected three bacterial species that had shown the highest accumulation of ROS in the presence of PSB2: *Streptococcus pyogenes*, *Streptococcus agalactiae*, and *Enterococcus faecalis*. It is important to mention that vitamin C does not affect the bacterial growth of these strains. We treated the bacteria with either PSB2 alone or a combination of PSB2 and vitamin C, after which we measured the ROS levels using the H_2_DCFDA probe. As a positive control, we used H_2_O_2_ instead of PSB2. As shown in Figure 7, vitamin C successfully reduced the formation of ROS in both the H_2_O_2_-treated positive control and in the samples treated with PSB2.

**Figure 8:**
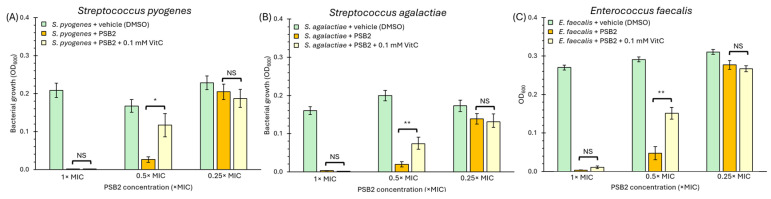
Vitamin C (VitC) affects bacterial growth in the presence of PSB2 for *Streptococcus pyogenes* (**A**), *Streptococcus agalactiae* (**B**), or *Enterococcus faecalis* (**C**). Bacteria were treated with the vehicle alone (DMSO), PSB2, or PSB2 + vitamin C (0.1 mM VitC). Bacterial growth was estimated according to OD_600_. 1 × MIC corresponds to the minimum inhibitory concentration of PSB2 (refer to Table 2). Furthermore, 0.5 × MIC corresponds to half of the MIC, while 0.25 × MIC corresponds to a quarter. Bars represent the standard error (SEM), n = 3. One-way ANOVA was performed as the statistical test. * *p* < 0.05, ** *p* < 0.01; NS = not significant.

Considering these results, the second step to determine the relative importance of ROS formation in the biocidal effect of PSB2, given that vitamin C strongly reduces ROS generation (Figure 7), was to repeat the experiment to determine the minimum inhibitory concentration (MIC) in the presence of vitamin C. If ROS generation is the primary biocidal mechanism of PSB2, we would expect vitamin C to completely negate this biocidal effect. Conversely, if PSB2 has an alternative antimicrobial mechanism that does not primarily involve ROS generation, we would observe that vitamin C either does not negate or only partially negates the biocidal effect of PSB2. In this context, we tested three Gram-positive bacteria exhibiting the highest ROS accumulation after PSB2 treatment, including *Streptococcus pyogenes*, *Streptococcus agalactiae*, and *Enterococcus faecalis*. As shown in Figure 8, vitamin C does not increase the MIC of PSB2. For this reason, we also plotted bacterial growth, inferred by optical density at 600 nm (OD_600_), to better understand the effect of ROS. Although the presence of vitamin C could not change the MIC, it can be observed that it increased bacterial growth when using half of the MIC with all of the strains tested. The partial improvement in bacterial growth in the presence of PSB2 due to the addition of vitamin C indicates that the ROS produced by PSB2 is secondary ROS and does not correspond to the primary source of PSB2’s biocidal effect. The observed reduction in ROS through vitamin C’s antioxidant properties supports the hypothesis that while PSB2 can induce ROS generation, this is not the primary mechanism through which it exerts its antimicrobial activity. Instead, the data suggest that PSB2 operates via an alternative pathway to inhibit bacterial growth. Further experiments are necessary to elucidate the exact mechanisms through which PSB2 exerts its biocidal effect, which will be crucial for understanding and potentially enhancing its application as an antimicrobial agent. 

## 3. Experimental and Theoretical Details

### 3.1. Materials and Instruments

All reagents were purchased from Sigma-Aldrich (St. Louis, MO, USA) and used without prior purification. FTIR (ATR) spectra were obtained using a Bruker Vector-22 FT-IR spectrophotometer (Bruker Corporation, Billerica, MA, USA). ^1^HNMR, ^13^CNMR, HHCOSY, DEPT-45 spectra, and D_2_O were recorded on a Bruker AVANCE 400 spectrometer (Bruker Corporation; Billerica, MA, USA) operating at 400 MHz and 25 °C, with samples dissolved in deuterated DMSO. Melting points were determined using a Stuart Scientific melting point apparatus SMP3 (UK) with open capillary tubes. Elemental analysis (CHNS) was conducted using a Thermo Flash 2000 Series (Thermo Fisher Scientific, Waltham, MA, USA) with a thermal conductivity detector (TCD). Thermal studies were performed with a Star System 1 thermogravimetric analyzer (TGA) at a heating rate of 10 °C min^−1^. Differential scanning calorimetry (DSC) measurements were executed using a Mettler Toledo Star System 822e (Mettler Toledo, Columbus, OH, USA) to determine the ligands’ glass transition temperature (Tg) at a heating rate of 10 °C min^−1^. TGA experiments were conducted under a nitrogen atmosphere.

Stock solutions of PSB1 to PSB4 (1.25 × 10^−3^ mol/L) were prepared and diluted with methanol in a glass flask for UV-Vis spectroscopy analysis. UV-Vis absorption spectra were recorded on an Agilent 8454 Diode-Array (Agilent Technologies, Santa Clara, CA, USA) spectrophotometer in aerated solutions in dichloromethane and DMSO. Molar absorptivity was determined according to the Lambert–Beer Law by measuring the absorbance at the maximum absorption of each band in each solvent using solutions of different concentrations of the base Schiff. A five-point calibration curve was established for each solution within the same measurement cell. This was accomplished by adding aliquots of 20 µL, 40 µL, 60 µL, 80 µL, or 100 µL of the stock solution to 3000 µL of the respective solvent being tested (dichloromethane and DMSO). This resulted in final concentrations of 8.278 × 10^−6^ M, 1.645 × 10^−5^ M, 2.451 × 10^−5^ M, 3.247 × 10^−5^ M, and 4.032 × 10^−5^ M, respectively. All measurements were conducted using a 1 cm quartz cuvette.

### 3.2. Procedure for Preparing Pyridine Schiff (PSB) Bases for This Study

PSB1 to PSB4 were synthesized through a direct reaction between the corresponding amino pyridine and hydroxy aldehyde compounds in a 1:1 molar ratio utilizing 20 mL of methanol as the solvent, following a previously described method [18,31]. The synthesis involved the condensation reaction between 3,4-diaminopyridine and 3,5-dibromo-2-hydroxybenzaldehyde for PSB1, 2-hydroxy-3,5-diiodobenzaldehyde for PSB2, 5-iodosalicylaldehyde for PSB3, and 5-chloro-3-iodosalicylaldehyde for PSB4. The reaction mixture was stirred for 24 h at room temperature without heating or an inert atmosphere. The resulting precipitate was filtered, washed with a 50:50 *v*/*v* mixture of ethanol and diethyl ether, and dried in a vacuum line. This synthesis procedure achieved yields exceeding 75% of the desired products.

#### 3.2.1. Synthesis of (E)-2-(((4-Aminopyridin-3-yl)imino)methyl)-4,6-dibromophenol (PSB1)

This process yielded a dark orange amorphous compound (yield 76%); decomposition point: 211–212 °C. FTIR (ATR, cm^−1^): 3483 and 3380 νNH_2_, 3068 νOH, 1626 νN=C, 1592 νC=C. ^1^HNMR (400 MHz, DMSO-d_6_, ppm): δ = 8.86 (s, 1H, H4), 8.05 (s, 1H, H3), 7.97 (d, *J* = 5.6 Hz, 1H, H1), 7.92 (m, 2H, H5 and H6), 6.68 (d, *J* = 5.5 Hz, 1H, H2), 6.25 (s, 2H, NH_2_). ^13^C-NMR (100 MHz, DMSO-d_6_, ppm): δ = 161.64 (C6: azomethine group), 157.03, 148.99, 148.18, 139.44, 137.62, 134.27, 131.14, 122.46, 112.05, 109.78. DEPT 45 (100 MHz, DMSO-d_6_, ppm): δ = 161.64 (C6: azomethine group), 148.18, 139.44, 137.62, 134.28, 109.79. Anal. Calcd. (%) for C_12_H_9_Br_2_N_3_O: C, 38.85; H, 2.44; N, 11.33; C/N = 3.43. Found (%): C, 40.19; H, 2.10; N, 11.36, C/N = 3.53.

#### 3.2.2. Synthesis of (E)-2-(((4-Aminopyridin-3-yl)imino)methyl)-4,6-diiodophenol (PSB2)

An orange amorphous compound was the final product (yield 75%); decomposition point: 189–190 °C. FTIR (ATR, cm^−1^): 3455 and 3353 νNH_2_, 3056 νOH, 1605 νN=C, 1592 νC=C. ^1^H-NMR (400 MHz, DMSO-d_6_, ppm): δ = 8.77 (s, 1H, H4), 8.15 (d, *J* = 2.5 Hz, 1H, H5), 8.06–8.00 (m, 2H, H3 and H6), 7.97 (d, *J* = 5.6 Hz, 1H, H1), 6.68 (d, *J* = 5.6 Hz, 1H, H2), 6.22 (s, 2H, NH_2_). ^13^C-NMR (100 MHz, DMSO-d_6_, ppm): δ = 161.90 (C6: azomethine group), 159.68, 148.91, 148.54, 148.17, 141.15, 139.60, 131.06, 122.02, 109.81, 88.54, 81.67. DEPT 45 (100 MHz, DMSO-d_6_, ppm): δ = 161.91 (C6: azomethine group), 148.54, 148.16, 141.16, 139.60, 109.81. Anal. Calcd. (%) for C_12_H_9_I_2_N_3_O: C, 30.99; H, 1.95; N, 9.04; C/N = 3.43. Found (%): C, 30.47; H, 1.55; N, 8.59, C/N = 3.54.

#### 3.2.3. Synthesis of (E)-2-(((4-Aminopyridin-3-yl)imino)methyl)-4-iodophenol (PSB3)

A light yellow amorphous compound was obtained (yield 79%); decomposition point: 163–164 °C. FTIR (ATR, cm^−1^): 3465 and 3298 νNH_2_, 3066 νOH, 1646 νN=C, 1590 νC=C. ^1^H-NMR (400 MHz, DMSO-d_6_, ppm): δ = 12.07 (s, 1H, OH), 8.73 (s, 1H, H4), 8.02 (d, *J* = 2.3 Hz, 1H, H5), 7.91 (s, 1H, H3), 7.87 (d, *J* = 5.4 Hz, 1H, H1), 7.60 (dd, *J* = 8.6 Hz, 2.3 Hz, 1H, H6), 6.75 (d, *J* = 8.7 Hz, 1H, H7), 6.57 (d, *J* = 5.5 Hz, 1H, H2), 5.98 (s, 2H, NH_2_). ^13^C-NMR (100 MHz, DMSO-d_6_, ppm): δ = 159.87 (C6: azomethine group), 159.61, 148.69, 148.34, 141.40, 139.66, 139.33, 132.17, 123.28, 119.70, 109.55, 81.34. DEPT 45 (100 MHz, DMSO-d_6_, ppm): δ = 159.87 (C6: azomethine group), 148.34, 141.40, 139.66, 139.33, 119.71, 109.56. Anal. Calcd. (%) for C_12_H_10_IN_3_O: C, 42.50; H, 2.97; N, 12.39; C/N = 3.43. Found (%): C, 42.18; H, 2.47; N, 11.90, C/N = 3.54.

#### 3.2.4. Synthesis of (E)-2-(((4-Aminopyridin-3-yl)imino)methyl)-4-chloro-6-iodophenol (PSB4)

An orange amorphous compound was the resulting product (yield 79%); decomposition point: 195–196 °C. FTIR (ATR, cm^−1^): 3425 and 3375 νNH_2_, 3284 νOH, 1650 νN=C, 1596 νC=C. ^1^H-NMR (400 MHz, DMSO-d_6_, ppm): δ = 8.72 (s, 1H, H4), 7.97 (s, 1H, H3), 7.93–7.85 (m, 2H, H1 and H5), 7.73 (d, *J* = 2.5 Hz, 1H, H6), 6.61 (d, *J* = 5.6 Hz, 1H, H2), 6.14 (s, 2H, NH_2_). ^13^C-NMR (100 MHz, DMSO-d_6_, ppm): δ = 161.88 (C6: azomethine group), 158.89, 148.86, 148.33, 140.77, 139.69, 132.23, 131.06, 123.45, 120.53, 109.82, 87.57. DEPT 45 (100 MHz, DMSO-d_6_, ppm): δ = 161.89 (C6: azomethine group), 148.33, 140.77, 139.69, 132.23, 109.82. Anal. Calcd. (%) for C_12_H_9_ClIN_3_O: C, 38.58; H, 2.43; N, 11.25; C/N = 3.43. Found (%): C, 38.64; H, 1.98; N, 10.86, C/N = 3.55.

### 3.3. DFT Calculations

All structural and electronic properties were determined using the Amsterdam Density Functional (ADF) code [25,28]. The molecular structures were fully optimized using the analytical energy gradient method developed by Verluis and Ziegler utilizing the hybrid B3LYP functional [29,30]. Standard Slater-type-orbital (STO) basis sets with triple-z quality double plus polarization functions (TZ2P) were applied to all atoms. Frequency analyses were conducted post-geometry optimization to confirm the minima and to compare the results with experimental infrared spectra.

Time-dependent density functional theory (TDDFT) was employed at the same theoretical level to compute excitation energies using the conductor-like screening model for realistic solvents (COSMO) [31,32]. Calculations were performed in three different solvents (dichloromethane, acetonitrile, and DMSO) to evaluate hydrogen bond stability and potential conformational changes influenced by solvent polarity. Additional calculations were carried out in the gas phase. The natural bond orbital (NBO) analysis was used to characterize the intramolecular hydrogen bond (IHB) energy [87,88].

### 3.4. Antimicrobial Activity

PSB1, PSB2, PSB3, and PSB4, along with some of their corresponding precursors—3,4-diaminopyridine, 3,5-dibromo-2-hydroxybenzaldehyde, 2-hydroxy-3,5-diiodobenzaldehyde—were evaluated for their in vitro growth-inhibitory activity (minimal inhibitory concentration, MIC) against various microorganisms. We tested Gram-negative bacteria, including *Salmonella enterica* subspecies *enterica* serovar Typhi strain STH2370 (*S.* Typhi) [89], and mutant derivatives of this parental strain, such as *S.* Typhi Δ*ompA* [90,91], *S.* Typhi Δ*yibP* [90], and *S.* Typhi Δ*rpoS* [36,92]. Additionally, we tested *Salmonella enterica* subspecies *enterica* serovar Typhimurium strain ATCC14028s and clinical strains from the Hospital Clínico de la Universidad de Chile, including *Escherichia coli* and *Morganella morganii*.

Furthermore, we tested non-sporulating, Gram-positive clinical bacteria from the Hospital Clínico de la Universidad de Chile, including *Streptococcus agalactiae*, *Streptococcus pyogenes*, *Enterococcus faecalis*, *Staphylococcus haemolyticus*, and three strains of *Staphylococcus aureus* (strain 2, strain 6, and strain 7). Finally, we evaluated the sporulating Gram-positive bacterium *Bacillus subtilis*.

The minimum inhibitory concentration (MIC) was determined using the broth dilution method described in [33]. The MIC is defined as the lowest concentration of the tested compounds at which no bacterial growth is observed after incubation [33]. Bacteria were cultured in Luria-Bertani broth (LB; Bacto tryptone, 10 g/L; Bacto yeast extract, 5 g/L; NaCl, 5 g/L) at 37 °C with shaking until reaching an OD_600_ of 1.4 (stationary phase). The bacterial cultures were then diluted to a 0.5 McFarland standard with Luria-Bertani broth before being seeded into a 96-well plate. Stock solutions of the corresponding pyridine Schiff base or their precursors were prepared in dimethyl sulfoxide (DMSO) and serially diluted (base 2) before being added to the bacterial cultures. The plates were incubated for 24 h at 37 °C. In indicated cases, 0.1 mM L-ascorbic acid (Vitamin C, Sigma-Aldrich) was added as an antioxidant.

### 3.5. Determination of Intracellular Reactive Oxygen Species (ROS)

Intracellular ROS levels were assessed using the 2′,7′-dichlorodihydrofluorescein diacetate (H_2_DCFDA) probe, following a previously outlined procedure with specific adaptations [34,35]. Briefly, cells were cultivated aerobically to an OD_600_ of approximately 0.6 in LB broth at 37 °C. The cells were then incubated with 10 μM H_2_DCFDA for 30 min. After incubation, the cells were washed and resuspended in phosphate-buffered saline (PBS) before exposure to 1.5 mM H_2_O_2_. Fluorescence emissions were monitored periodically using a Biotech Synergy microplate (Agilent Technologies, Santa Clara, CA, USA) reader (excitation at 480 nm, emission at 520 nm). Fluorescence readings were normalized against protein concentrations. This experiment was conducted in biological triplicate.

### 3.6. Statistical Analysis

All data values are presented as the mean ± standard error (SEM) from three biological replicates, including three technical replicates. Statistical analysis was performed using Kruskal–Wallis and Dunn as post hoc tests.

## 4. Conclusions

This study contributes to the field of non-metallic antimicrobial agents by successfully synthesizing and characterizing a novel series of halogen-substituted pyridine Schiff bases. Through a comprehensive physicochemical analysis, including elemental, spectroscopic, and quantum theoretical studies, the presence of intramolecular hydrogen bonding was confirmed to contribute to the structural stability of these compounds. A major advantage of this work lies in the selective biocidal activity exhibited by these compounds, particularly against Gram-positive bacteria, which presents a potentially promising solution to the growing challenge of antibiotic resistance in such pathogens.

This study highlights the crucial influence of halogen substitution on the phenolic ring in enhancing antimicrobial efficacy, with PSB2 emerging as the most potent compound. This differential efficacy underscores the importance of substituents’ electronic and steric effects in designing therapeutically relevant compounds. Another key advantage is the discovery that, unlike many traditional biocides, PSB2 operates through mechanisms independent of reactive oxygen species (ROS) generation, indicating alternative antimicrobial pathways. This is particularly advantageous as it opens new avenues for developing antimicrobial agents with innovative modes of action, which could be critical in overcoming resistance mechanisms employed by bacteria against conventional therapies.

In conclusion, this study not only introduces new compounds with potential antimicrobial applications but also provides deeper insights into how structural modifications can enhance the bioactivity of Schiff bases. This lays the groundwork for the future development of more effective and stable antimicrobial agents with potential applications in clinical and pharmaceutical fields.

## Figures and Tables

**Table 1:** Donor–acceptor interaction analysis based on NBO calculations for PSBs.

Pyridine Schiff Base	Donor	Acceptor	ΔE (kcal/mol)
PSB1	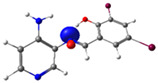 N4 (LP)	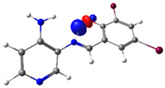 O1-H2 (BD*)	10.3 (IHB)
	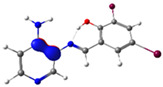 C7-C8 (BD)	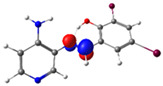 N4-C13 (BD*)	11.6
	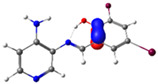 C14-C15 (BD)		21.6
PSB2	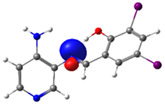 N4 (LP)	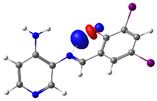 O1-H2 (BD*)	10.0 (IHB)
	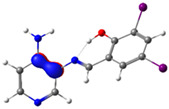 C7-C8 (BD)	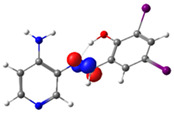 N4-C13 (BD*)	10.1
	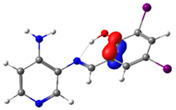 C14-C15 (BD)		21.7
PSB3	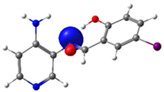 N4 (LP)	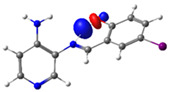 O1-H2 (BD*)	8.0 (IHB)
	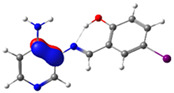 C7-C8 (BD)	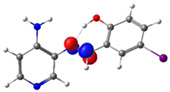 N4-C13 (BD*)	9.2
	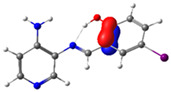 C14-C15 (BD)		22.3
PSB4	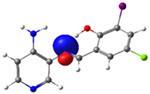 N4 (LP)	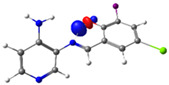 O1-H2 (BD*)	10.0 (IHB)
	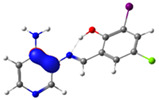 C7-C8 (BD)	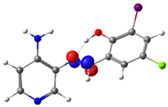 N4-C13 (BD*)	10.4
	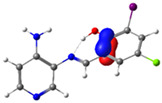 C14-C15 (BD)		21.7

LP: lone pair; BD: bonding; BD*: antibonding. For atom numbering, see Figure S40.

**Table 2:** Determination of Gram-positive bacteria’s minimal inhibitory concentration of pyridine Schiff bases (PSB1 to PSB4).

Species	Precursor 1 ^1^	MIC Precursor 1 (µM)	Precursor 2 ^2^	MIC Precursor 2 (µM)	Pyridine Schiff Base	MIC Pyridine Schiff Base (µM)
*Bacillus subtilis*	3,5-dibromo-2-hydroxybenzaldehyde	No effect	3,4-diaminopyridine	No effect	PSB1	25.3 ± 0.0
	2-hydroxy-3,5-diiodobenzaldehyde	6.3 ± 0.0	3,4-diaminopyridine	No effect	PSB2	5.7 ± 0.5
	5-iodosalicylaldehyde	Not determined	3,4-diaminopyridine	No effect	PSB3	No effect
	5-chloro-3-iodosalicylaldehyde	Not determined	3,4-diaminopyridine	No effect	PSB4	No effect
*Streptococcus agalactiae*	3,5-dibromo-2-hydroxybenzaldehyde	17.3 ± 2.3	3,4-diaminopyridine	No effect	PSB1	11.8 ± 0.7
	2-hydroxy-3,5-diiodobenzaldehyde	3.9 ± 0.5	3,4-diaminopyridine	No effect	PSB2	2.6 ± 0.2
	5-iodosalicylaldehyde	Not determined	3,4-diaminopyridine	No effect	PSB3	25.3 ± 0.0
	5-chloro-3-iodosalicylaldehyde	Not determined	3,4-diaminopyridine	No effect	PSB4	11.8 ± 0.7
*Streptococcus pyogenes*	3,5-dibromo-2-hydroxybenzaldehyde	9.4 ± 1.1	3,4-diaminopyridine	25.3 ± 0.0	PSB1	11.8 ± 0.7
	2-hydroxy-3,5-diiodobenzaldehyde	3.4 ± 0.7	3,4-diaminopyridine	25.3 ± 0.0	PSB2	1.3 ± 0.2
	5-iodosalicylaldehyde	Not determined	3,4-diaminopyridine	25.3 ± 0.0	PSB3	25.3 ± 0.0
	5-chloro-3-iodosalicylaldehyde	Not determined	3,4-diaminopyridine	25.3 ± 0.0	PSB4	6.3 ± 0.0
*Enterococcus faecalis*	3,5-dibromo-2-hydroxybenzaldehyde	50.5 ± 0.0	3,4-diaminopyridine	50.5 ± 0.0	PSB1	50.5 ± 0.0
	2-hydroxy-3,5-diiodobenzaldehyde	10.2 ± 1.1	3,4-diaminopyridine	50.5 ± 0.0	PSB2	5.3 ± 0.3
	5-iodosalicylaldehyde	Not determined	3,4-diaminopyridine	50.5 ± 0.0	PSB3	50.5 ± 0.0
	5-chloro-3-iodosalicylaldehyde	Not determined	3,4-diaminopyridine	50.5 ± 0.0	PSB4	50.5 ± 0.0
*Staphylococcus aureus* strain 2	3,5-dibromo-2-hydroxybenzaldehyde	12.6 ± 0.0	3,4-diaminopyridine	No effect	PSB1	12.6 ± 0.0
	2-hydroxy-3,5-diiodobenzaldehyde	6.3 ± 0.0	3,4-diaminopyridine	No effect	PSB2	3.2 ± 0.0
	5-iodosalicylaldehyde	Not determined	3,4-diaminopyridine	No effect	PSB3	No effect
	5-chloro-3-iodosalicylaldehyde	Not determined	3,4-diaminopyridine	No effect	PSB4	11.8 ± 0.7
*Staphylococcus aureus* strain 6	3,5-dibromo-2-hydroxybenzaldehyde	11.8 ± 0.7	3,4-diaminopyridine	50.5 ± 0.0	PSB1	12.6 ± 0.0
	2-hydroxy-3,5-diiodobenzaldehyde	6.3 ± 0.0	3,4-diaminopyridine	50.5 ± 0.0	PSB2	3.5 ± 0.3
	5-iodosalicylaldehyde	Not determined	3,4-diaminopyridine	50.5 ± 0.0	PSB3	6.3 ± 0.0
	5-chloro-3-iodosalicylaldehyde	Not determined	3,4-diaminopyridine	50.5 ± 0.0	PSB4	11.0 ± 1.0
*Staphylococcus aureus* strain 7	3,5-dibromo-2-hydroxybenzaldehyde	12.6 ± 0.0	3,4-diaminopyridine	50.5 ± 0.0	PSB1	12.6 ± 0.0
	2-hydroxy-3,5-diiodobenzaldehyde	6.3 ± 0.0	3,4-diaminopyridine	50.5 ± 0.0	PSB2	3.2 ± 0.0
	5-iodosalicylaldehyde	Not determined	3,4-diaminopyridine	50.5 ± 0.0	PSB3	50.5 ± 0.0
	5-chloro-3-iodosalicylaldehyde	Not determined	3,4-diaminopyridine	50.5 ± 0.0	PSB4	11.8 ± 0.7
*Staphylococcus haemolyticus*	3,5-dibromo-2-hydroxybenzaldehyde	No effect	3,4-diaminopyridine	No effect	PSB1	No effect
	2-hydroxy-3,5-diiodobenzaldehyde	12.6 ± 0.0	3,4-diaminopyridine	No effect	PSB2	6.3 ± 0.0
	5-iodosalicylaldehyde	Not determined	3,4-diaminopyridine	No effect	PSB3	No effect
	5-chloro-3-iodosalicylaldehyde	Not determined	3,4-diaminopyridine	No effect	PSB4	25.3 ± 0.0

^1^ Precursor 1 is used to synthesize the corresponding pyridine Schiff base (phenol aldehyde precursors), ^2^ Precursor 2 corresponds to 3,4-diaminopyridine in all cases.

## Data Availability

Data are contained within the article and Supplementary Materials.

## Supplementary Materials

The following supporting information can be downloaded at: https://www.mdpi.com/article/10.3390/molecules29194726/s1, Table S1: Characteristic constants of non-metal pyridine Schiff bases (PSBs); Table S2: Calculated frequencies (cm^−1^) for PSB1 to PSB4; Table S3. Proton assignments (in ppm) of PSBs; Table S4: Experimental maximum wavelengths (λ), molar extinction coefficients (ε) and quantum yield (φ) of PSBs; Table S5: Calculated wavelengths (nm), oscillator strengths (f) and corresponding transition of PSBs in DMSO; Table S6: Molecular orbitals involved in the electronic transition of absorption located at 378–389 nm for PSBs; Table S7: Optimized geometry parameters for PBs in the ground state; Figure S1: Mass spectra of PSB1; Figure S2: Mass spectra of PSB2; Figure S3: Mass spectra of PSB3; Figure S4: Mass spectra of PSB4; Figure S5: TGA of PSB1 in inert atmosphere (N2); Figure S6: TGA of PSB2 in inert atmosphere (N2); Figure S7: TGA of PSB3 in inert atmosphere (N2); Figure S8: TGA of PSB4 in inert atmosphere (N2); Figure S9: ATR of PSB1; Figure S10: ATR of PSB2; Figure S11: ATR of PSB3; Figure S12: ATR of PSB4; Figure S13: Vibrational spectrum analysis of PSB1; Figure S14: Vibrational spectrum analysis of PSB2; Figure S15: Vibrational spectrum analysis of PSB3; Figure S16: Vibrational spectrum analysis of PSB4; Figure S17: Arbitrary numbering of protons for (A) PSB1, PSB2 and PSB4, and (B) PSB3; Figure S18: ^1^H-NMR of PSB1 in DMSO-d_6_; Figure S19: ^1^H-NMR of PSB2 in DMSO-d_6_; Figure S20: ^1^H-NMR of PSB3 in DMSO-d_6_; Figure S21: ^1^H-NMR of PSB4 in DMSO-d_6_; Figure S22: D_2_O exchange of PSB1 in DMSO-d_6_; Figure S23: D_2_O exchange of PSB2 in DMSO-d_6_; Figure S24: D_2_O exchange of PSB3 in DMSO-d_6_; Figure S25: D_2_O exchange of PSB4 in DMSO-d_6_; Figure S26: HHCOSY of PSB1 in DMSO-d_6_; Figure S27: HHCOSY of PSB2 in DMSO-d_6_; Figure S28: HHCOSY of PSB3 in DMSO-d_6_; Figure S29: HHCOSY of PSB4 in DMSO-d_6_; Figure S30: Numbering atoms used in carbon assignments for PSBs; Figure S31: ^13^C-NMR of PSB1 in DMSO-d_6_; Figure S32: ^13^C-NMR of PSB2 in DMSO-d_6_; Figure S33: ^13^C-NMR of PSB3 in DMSO-d_6_; Figure S34: ^13^C-NMR of PSB4 in DMSO-d_6_.; Figure S35: DEPT 45 of PSB1 in DMSO-d_6_.; Figure S36: DEPT 45 of PSB2 in DMSO-d_6_; Figure S37: DEPT 45 of PSB3 in DMSO-d_6_; Figure S38: DEPT 45 of PSB4 in DMSO-d_6_; Figure S39: Normalized emission spectra recorded in dichloromethane (DCM) of PSBs at room temperature; Figure S40: Numbering atoms of PSBs with the different substitutions (black color) in the phenolic ring.
