# Supplementary material for: Synthesis, Physicochemical Characterization, and Antimicrobial Evaluation of Halogen-Substituted Non-Metal Pyridine Schiff Bases
Source: Molecules. 2024 Oct 6;29(19):4726. doi: 10.3390/molecules29194726 (PMC11477791; doi:10.3390/molecules29194726)
Supplement: Supplementary file 1 [file molecules-29-04726-s001.zip › molecules-3187002-supplementary.pdf]

Supplementary Material

1. Supplementary Tables

Table S 1. Characteristic constants of non-metal pyridine Schiff bases (PSBs).

Schiff base	MF	MW (g/mol)	Yield (%)	Solid color
PSB1	$C_{12}H_9Br_2N_3O$	371.03	76	Orange
PSB2	$C_{12}H_9I_2N_3O$	465.03	75	Orange
PSB3	$C_{12}H_{10}IN_3O$	339.13	79	Yellow
PSB4	$C_{12}H_9ClIN_3O$	373.58	79	Orange

MF: Molecular formula

MW: Molecular weight

Table S 2. Calculated frequencies (cm^{-1}) for PSB1 to PSB4.

Schiff base	νNH_2(as)	νNH_2(s)	νOH	NCH (azomethine)	νC=N (azomethine)
PSB1	3715	3648	3186	3065	1671
PSB2	3712	3646	3182	3061	1671
PSB3	3710	3613	3278	3056	1672
PSB4	3712	3646	3186	3062	1671

as: asymmetric

s: symmetric

Table S 3. Proton assignments (in ppm) of PSBs.

Proton	PSB1 *	PSB2	PSB3	PSB4
H1	7.97	7.97	7.87	7.93-7.85
H2	6.68	6.68	6.57	6.61
H3	8.05	8.06-8.00	7.91	7.97
H4	8.86	8.77	8.73	8.72
H5	7.92	8.15	8.02	7.93-7.85
H6	7.92	8.06-8.00	7.60	7.73
H7	–	–	6.75	–

*For the arbitrary numbering of protons, see Figure S , respectively.

Table S 4. Experimental maximum wavelengths (λ), molar extinction coefficients (ε) and quantum yield (ϕ) of PSBs.

Schiff Base	Solvent	λ abs (nm)	λ em (nm)	ϕ	ε (L/mol×cm)
PSB1	DMSO	259			15488
		380	478	0.00215	7774
	DCM	235			9325
		370	540	0.00026	8285
PSB2	DMSO	259			25282
		379	442	0.00359	11527
	DCM	234			15115
		372	550	0.00035	13398
PSB3	DMSO	259			21311
		372	444	0.00342	10617
	DCM	234			14074
		368	534	0.00023	12233
PSB4	DMSO	259			25595
		377	449	0.00321	14003
	DCM	233			17645
		370	547	0.00032	16517

abs: absorption

em: emission

DMSO: dimethyl sulfoxide

DCM: dichloromethane

Table S 5. Calculated wavelengths (nm), oscillator strengths (f) and corresponding transition of PSBs in DMSO.

Schiff base	λ (nm)	f	Transition	Assignment
PSB1	389	0.563	H → L (98%)	π→π*
	239	0.324	H → L+4 (44%)	π→π*
PSB2	386	0.493	H → L (95%)	π→π*
	224	0.352	H-8 → L (48%)	π→π*/n→π*
PSB3	378	0.491	H → L (95%)	π→π*
	244	0.414	H-6 → L (52%)	π→π*/n→π*
PSB4	386	0.528	H → L (98%)	π→π*
	220	0.526	H-2 → L+2 (56%)	π→π*/n→π*

Table S 6. Molecular orbitals involved in the electronic transition of absorption located at 378-389 nm for PSBs

Schiff Base	HOMO	LUMO
PSB1	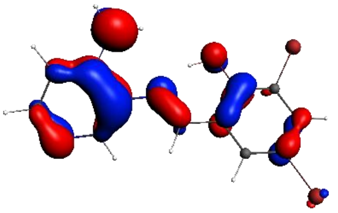	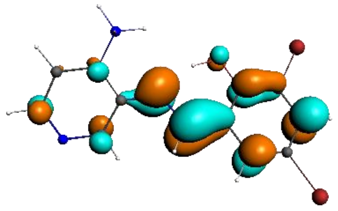
PSB2	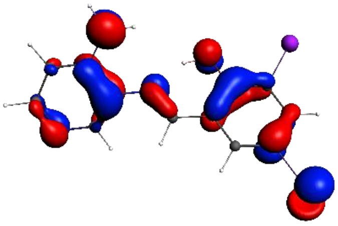	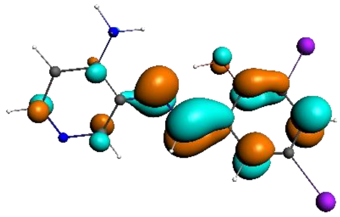
PSB3	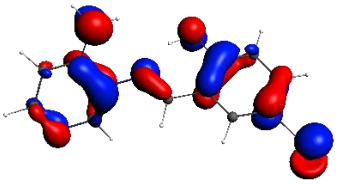	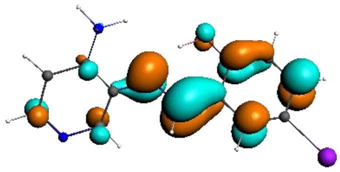
PSB4	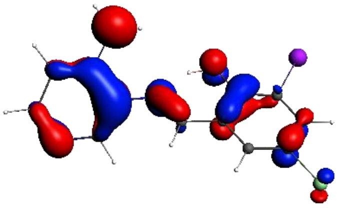	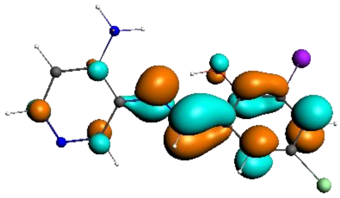

Table S 7. Optimized geometry parameters for PBs in the ground state

**Bond length (Å)**				
**Parameters**	**PSB1**	**PSB2**	**PSB3**	**PSB4**
C=N(azomethine)	1.285	1.285	1.286	1.285
C-O	1.337	1.337	1.344	1.338
O-H	0.993	0.993	0.989	0.993
$H \cdots N_4$	1.736	1.738	1.785	1.739
**Bond angle (°)**				
**Parameters**	**PSB1**	**PSB2**	**PSB3**	**PSB4**
$C_7N_4{=}C_{13}C_{14}$	175.59	175.81	175.53	175.20
$C_{13}C_{14}C_{15}O$	-0.41	0.22	0.63	0.49
$C_{14}C_{15}OH$	-1.64	0.12	0.24	-0.44
$COH \cdots N_4$	6.00	3.60	2.97	3.73

2. Supplementary Figures

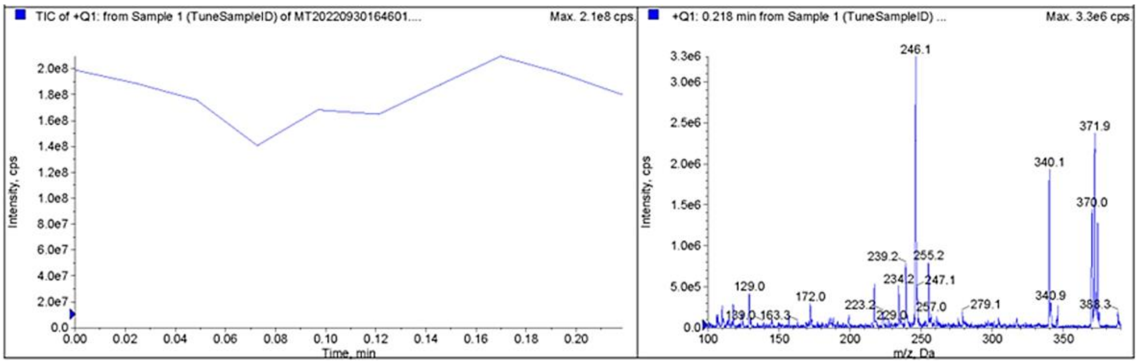

Figure S 1. Mass spectra of PSB1.

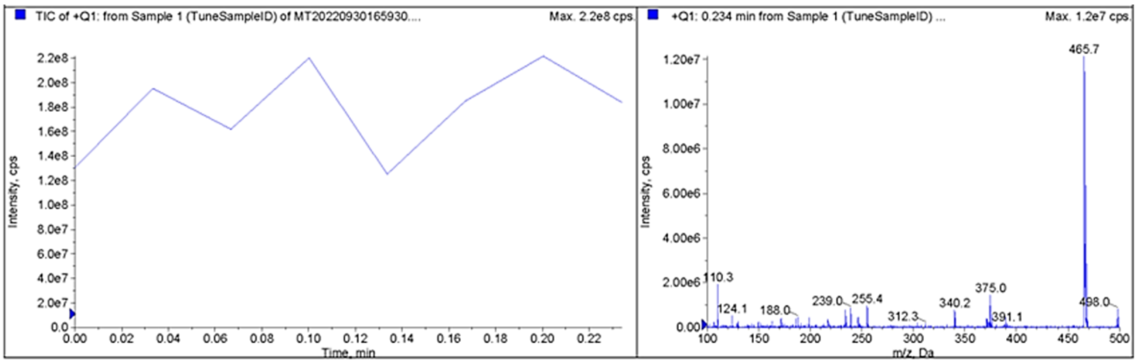

Figure S 2. Mass spectra of PSB2.

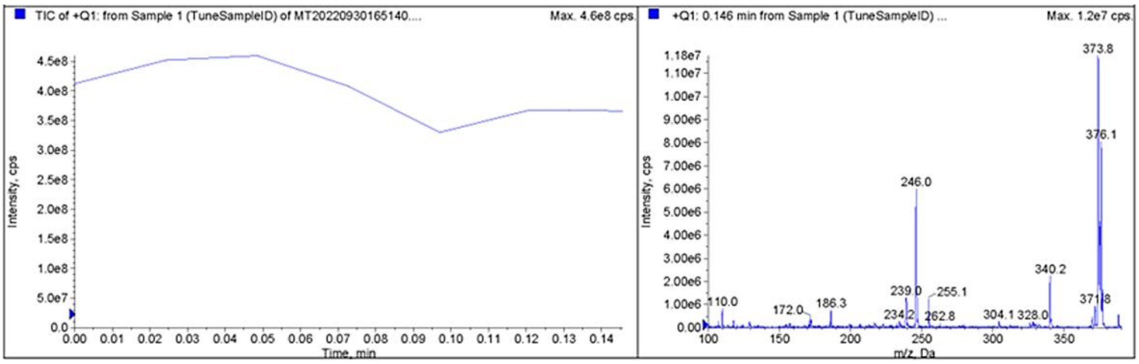

Figure S 3. Mass spectra of PSB3.

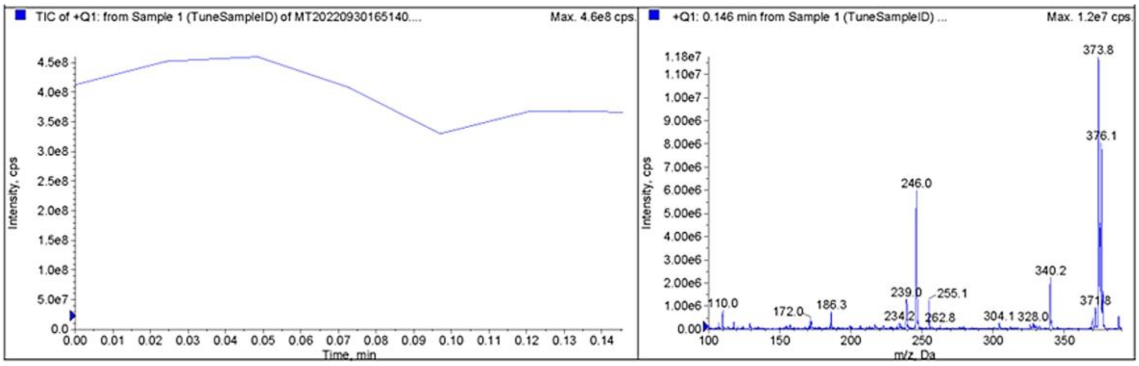

Figure S 4. Mass spectra of PSB4.

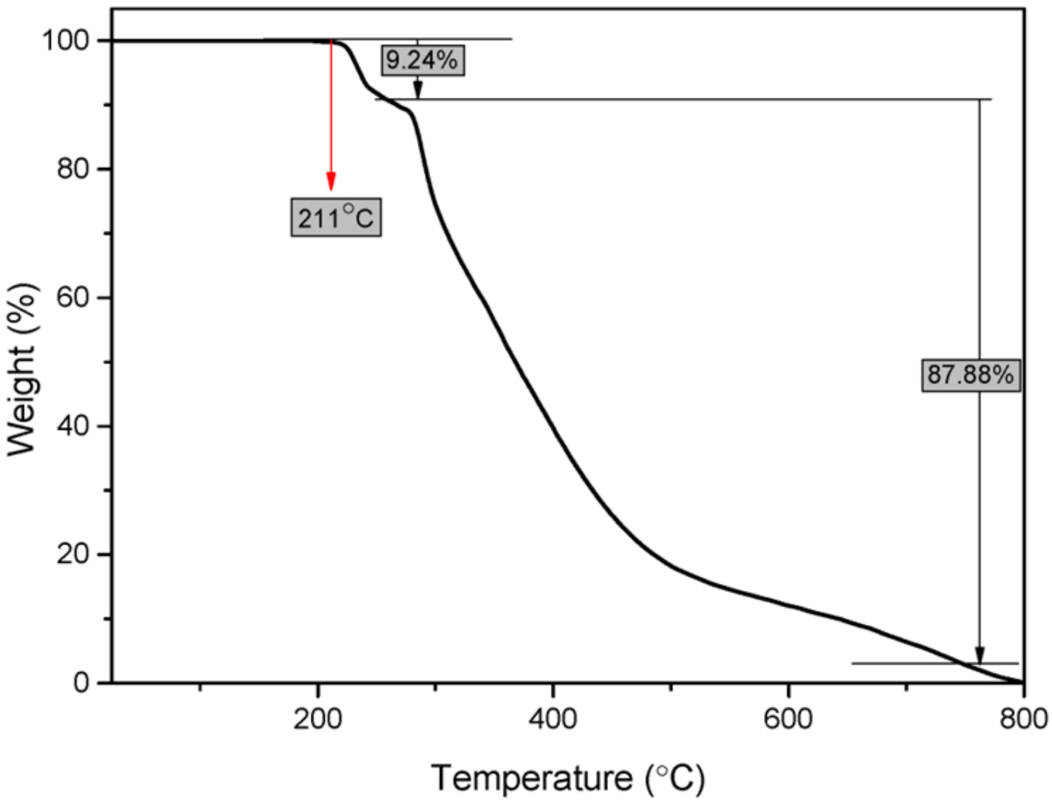

Figure S 5. TGA of PSB1 in inert atmosphere (N_2).

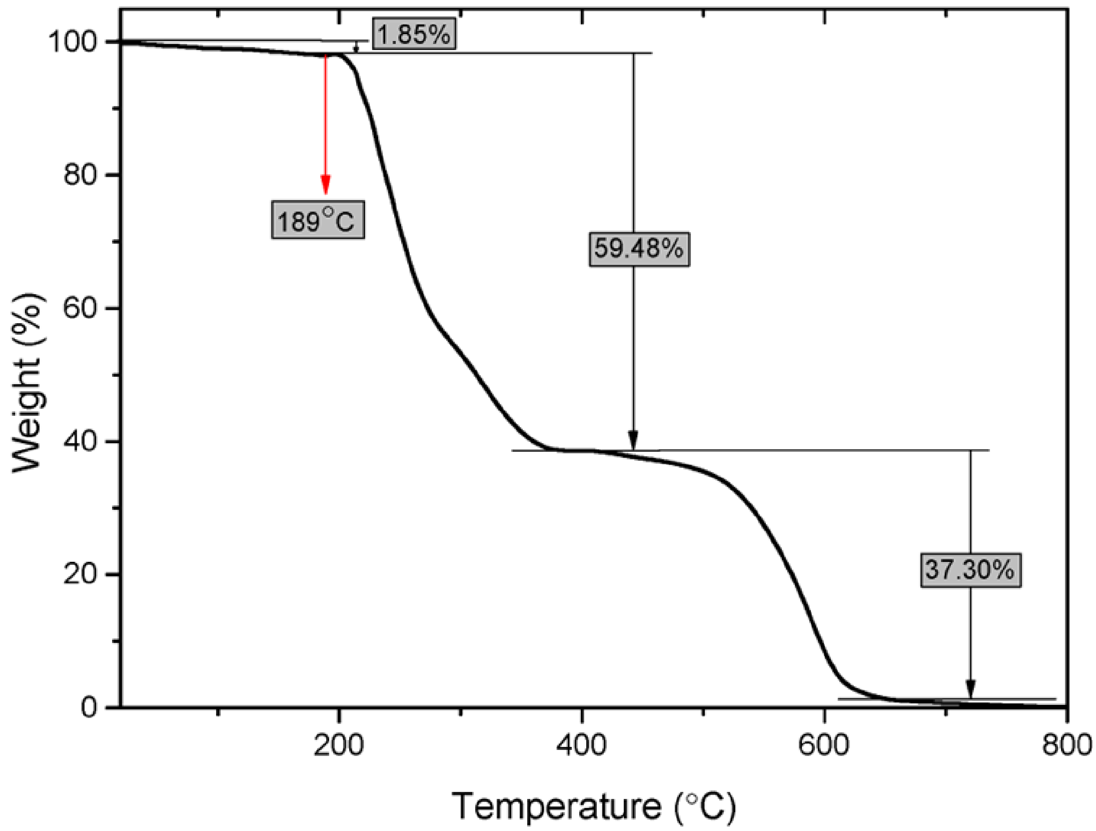

Figure S 6. TGA of PSB2 in inert atmosphere (N_2).

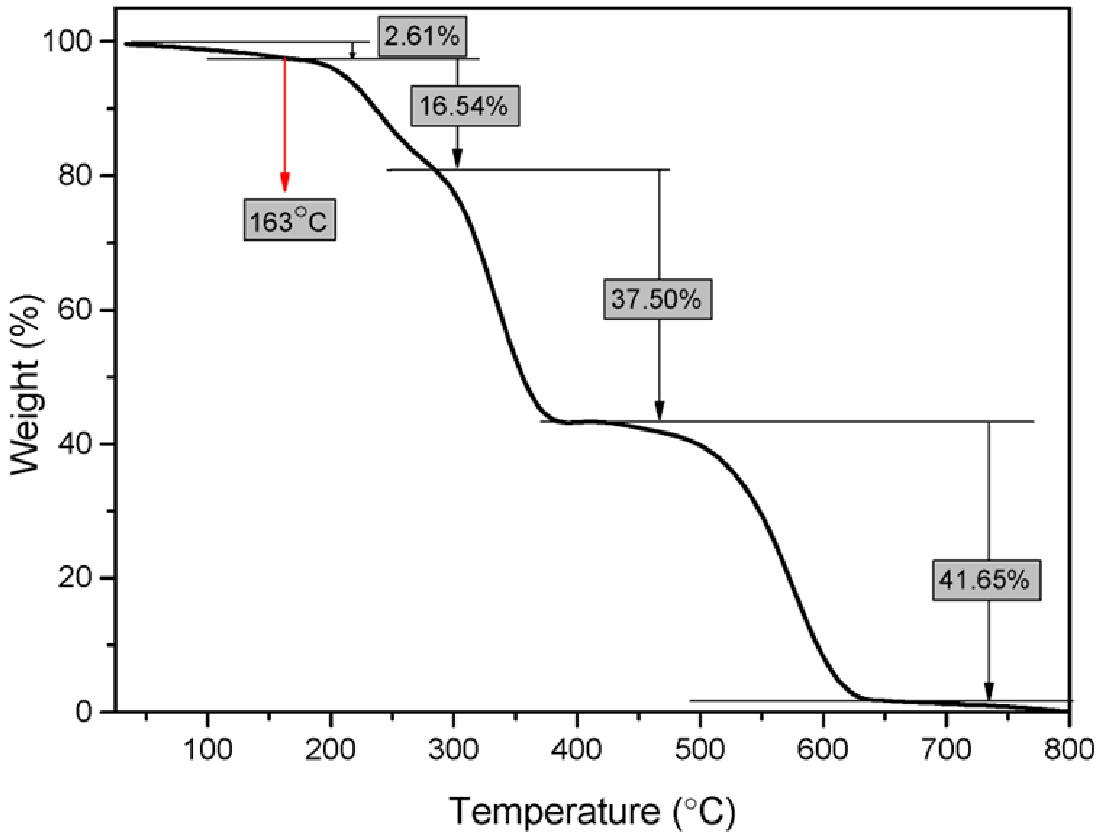

Figure S 7. TGA of PSB3 in inert atmosphere (N_2).

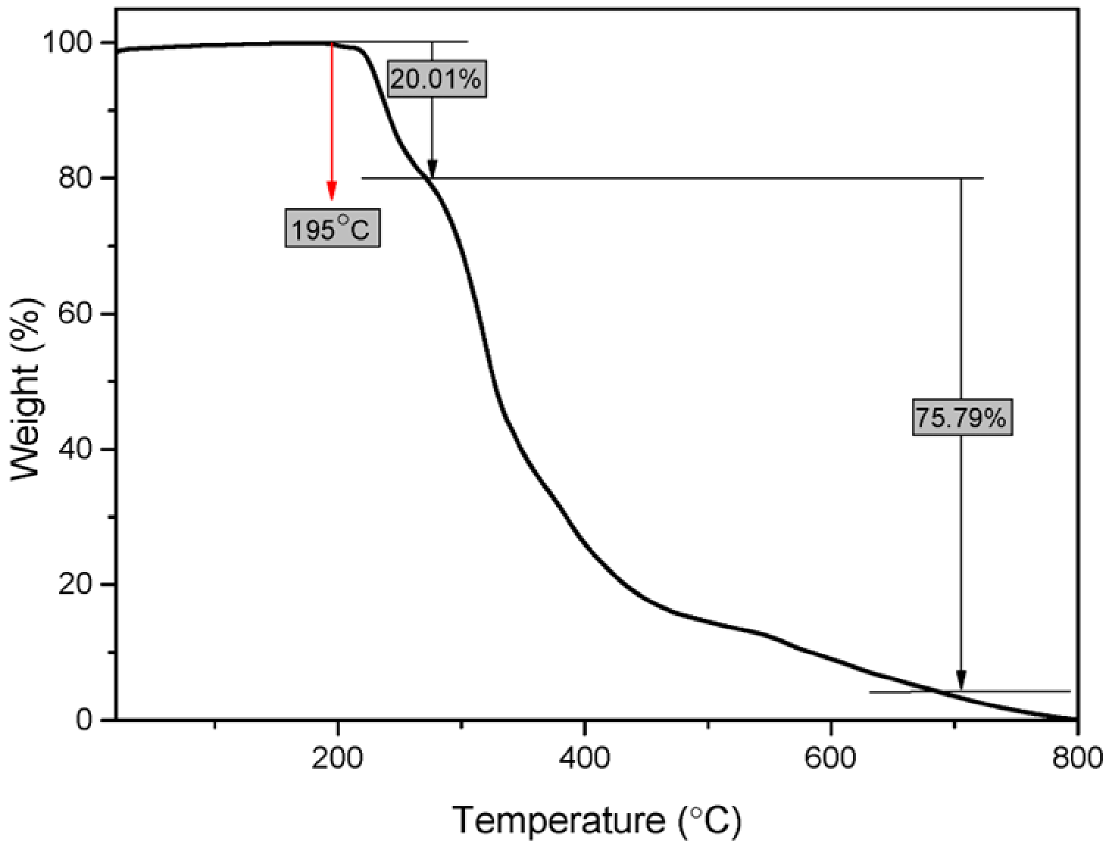

Figure S 8. TGA of PSB4 in inert atmosphere (N_2).

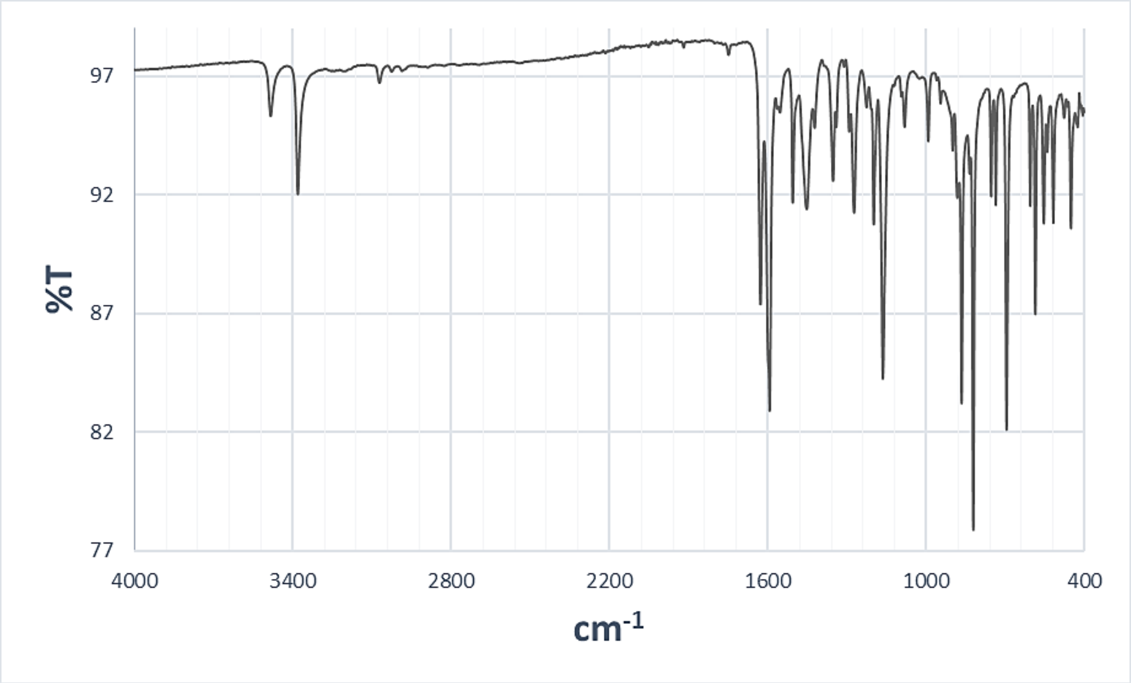

Figure S 9. ATR of PSB1.

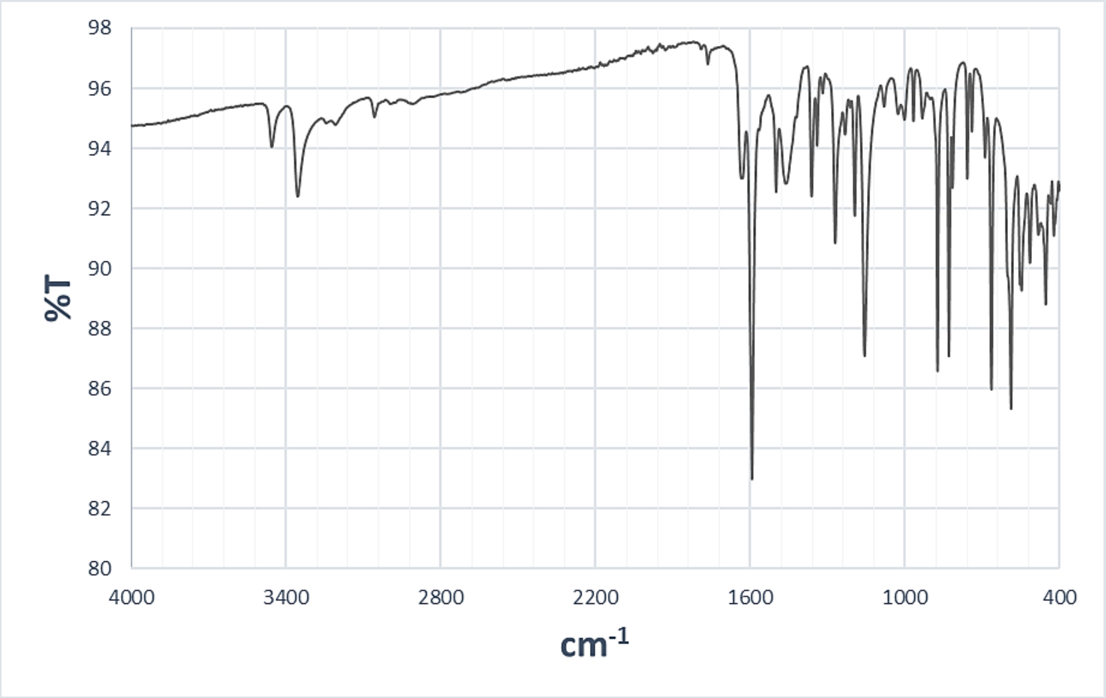

Figure S 10. ATR of PSB2.

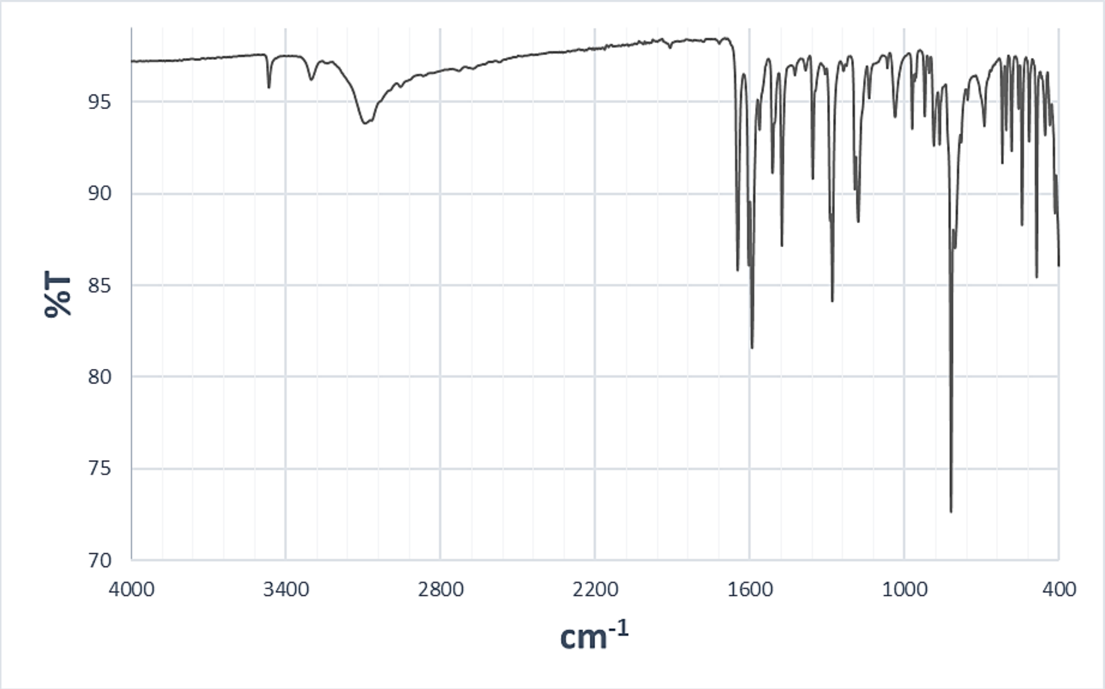

Figure S 11. ATR of PSB3.

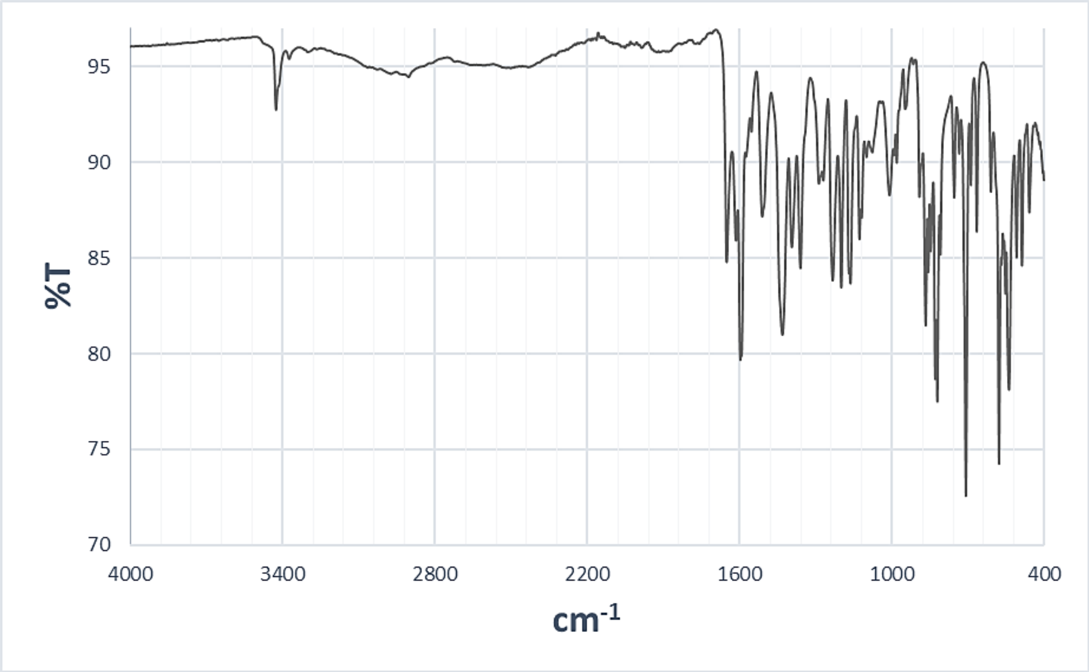

Figure S 12. ATR of PSB4.

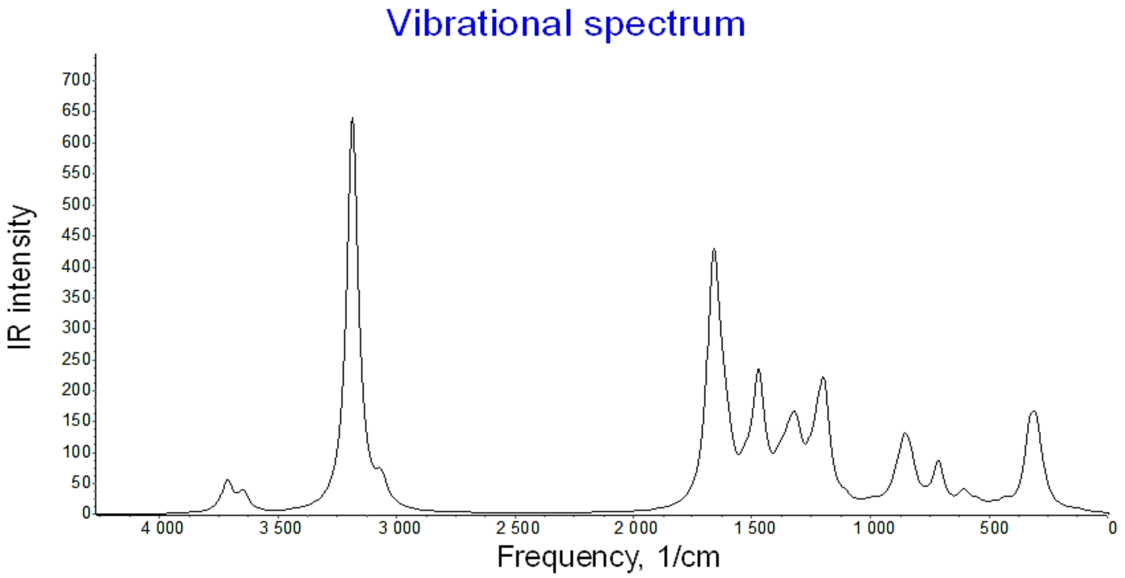

Figure S 13. Vibrational spectrum analysis of PSB1.

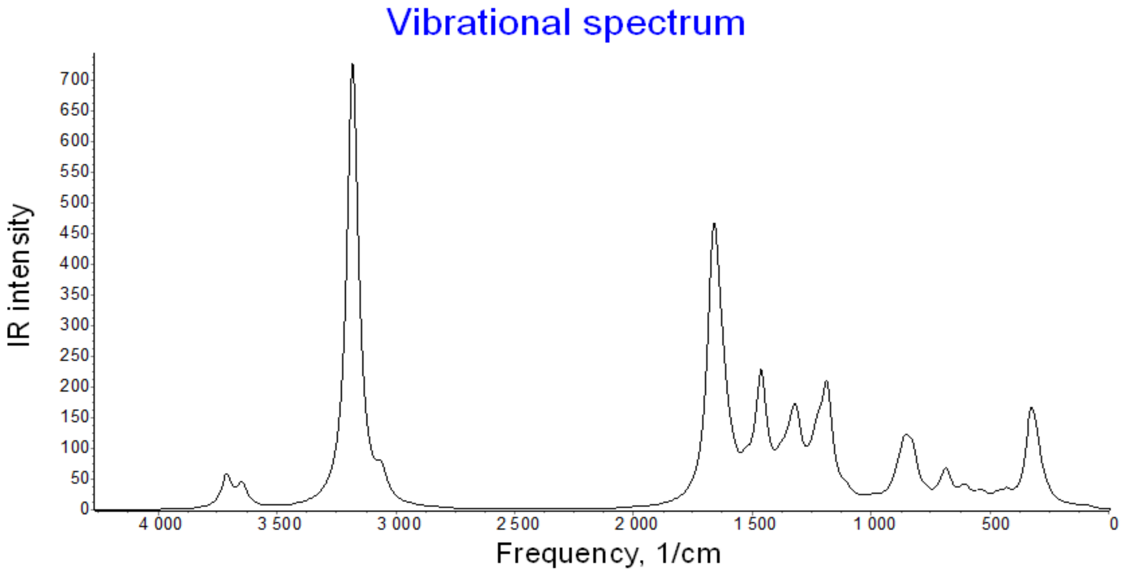

Figure S 14. Vibrational spectrum analysis of PSB2.

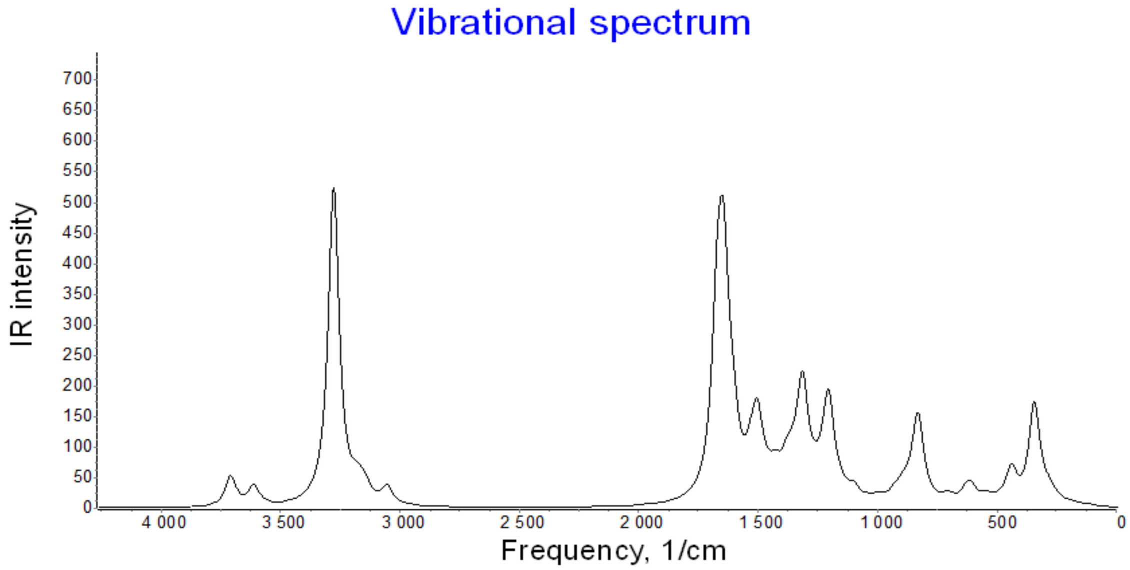

Figure S 15. Vibrational spectrum analysis of PSB3.

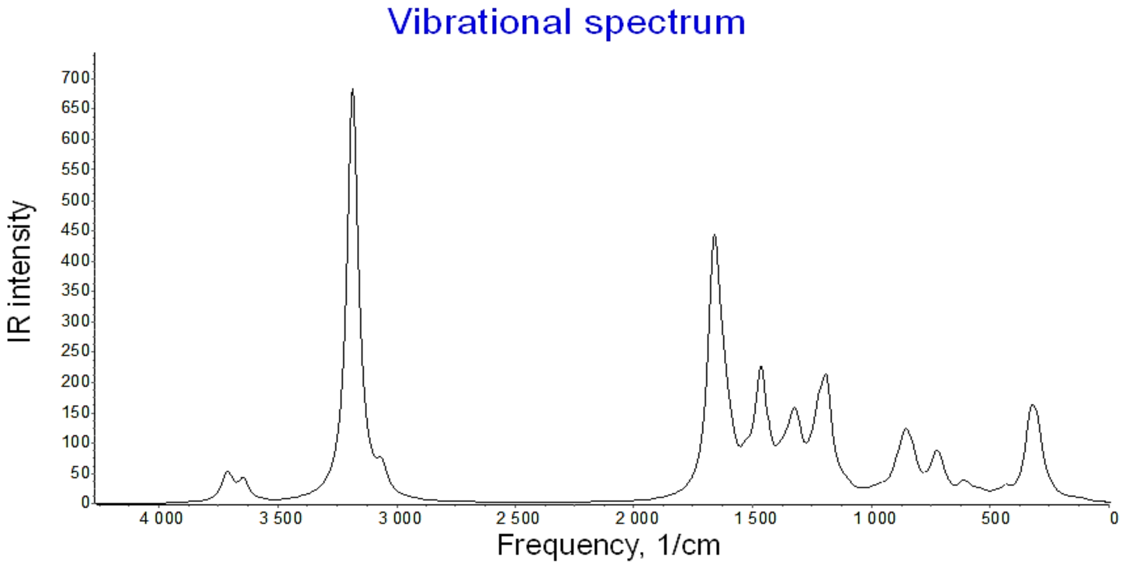

Figure S 16. Vibrational spectrum analysis of PSB4

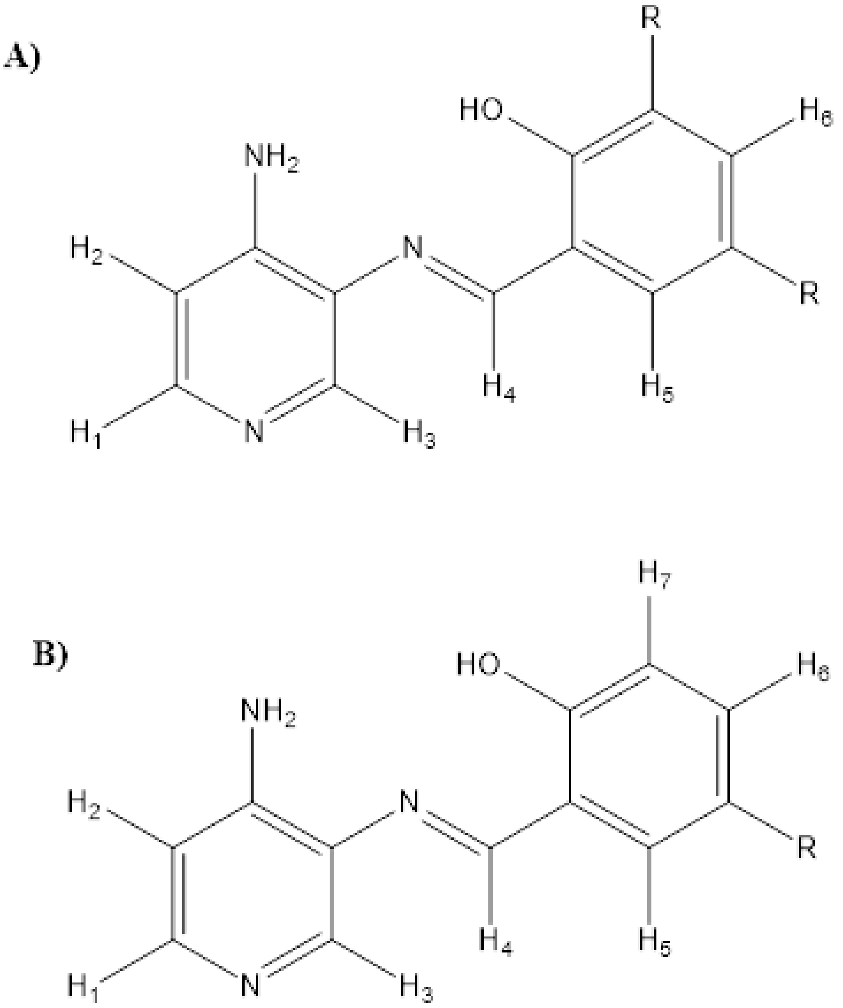

Figure S 17. Arbitrary numbering of protons for A) PSB1, PSB2 and PSB4, and B) PSB3

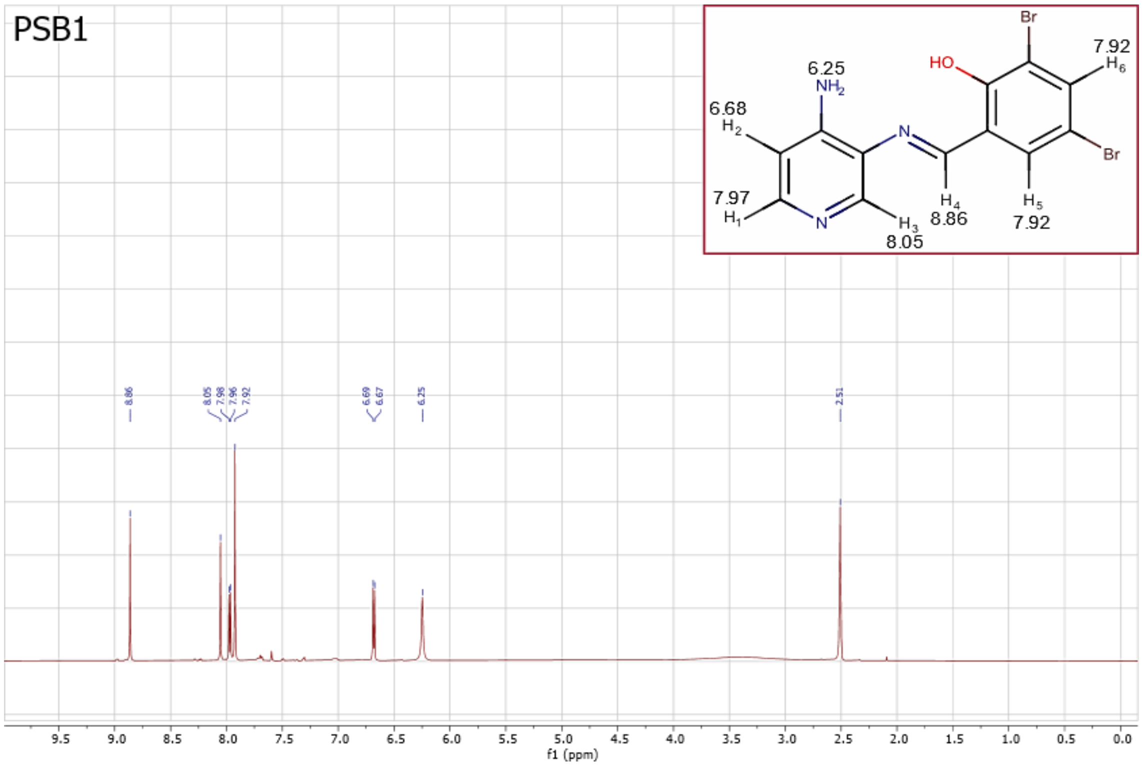

Figure S 18. ^{1}H-NMR of PSB1 in DMSO-d_6.

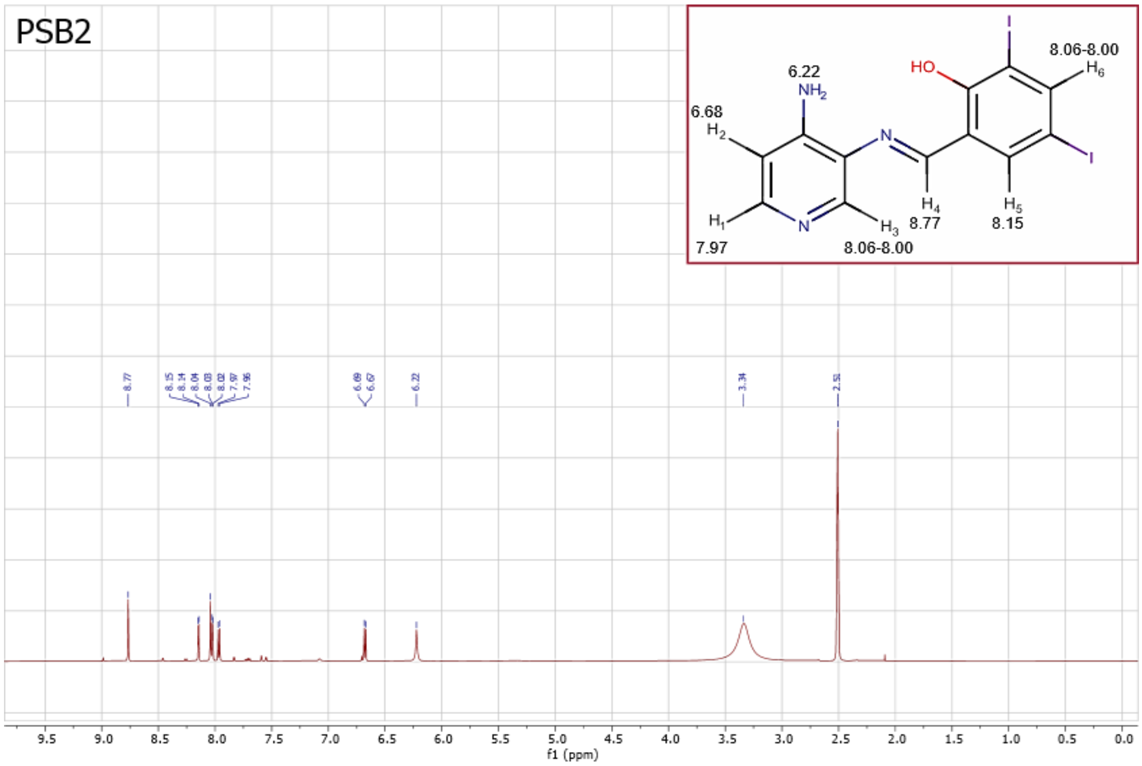

Figure S 19. ^{1}H-NMR of PSB2 in DMSO-d_6.

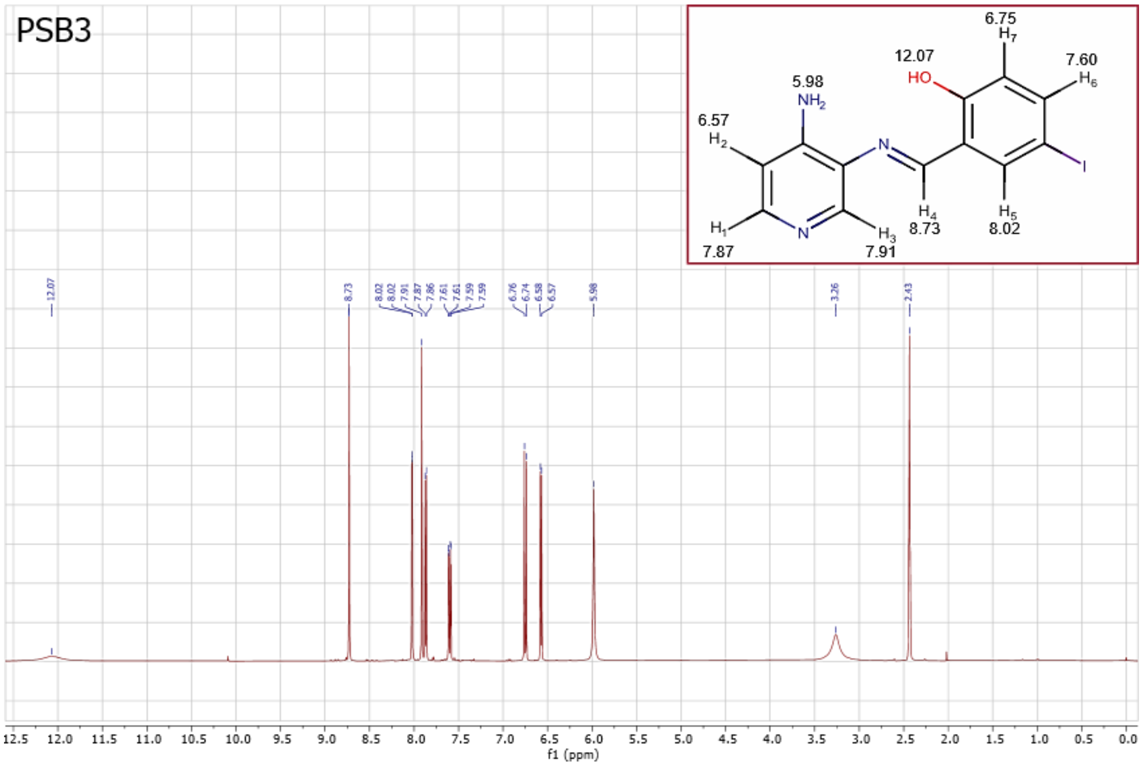

Figure S 20. ^{1}H-NMR of PSB3 in DMSO-d_6.

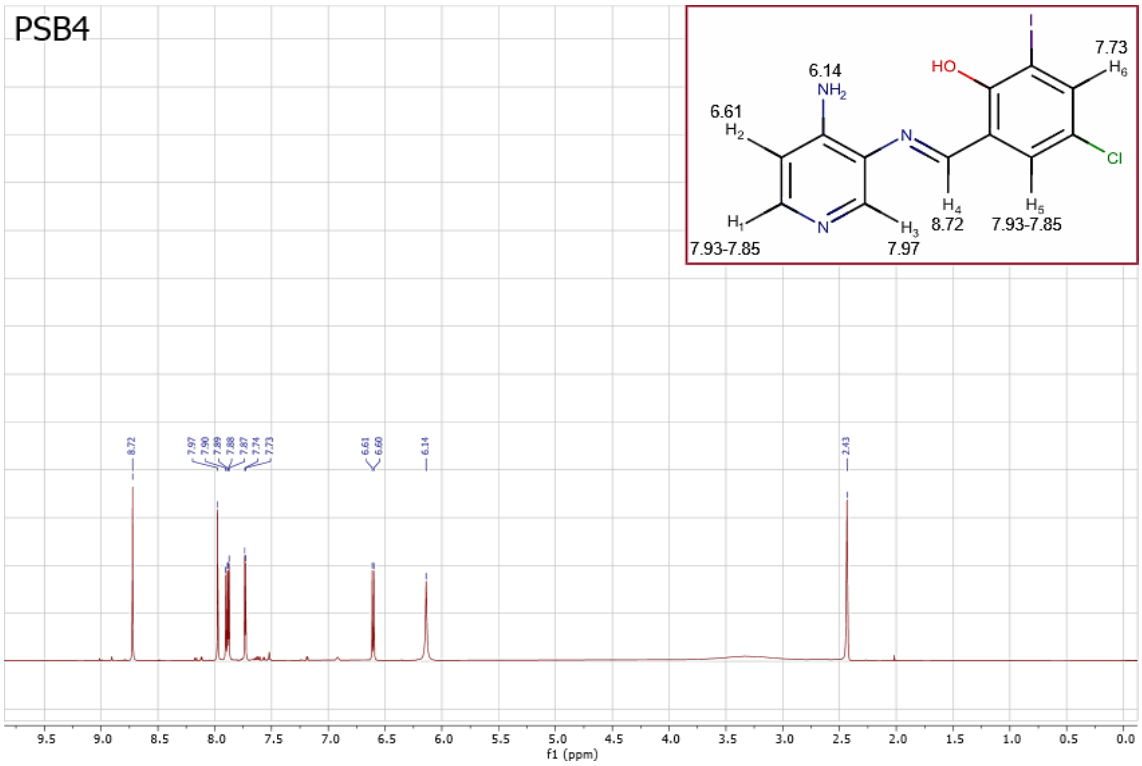

Figure S 21. ^{1}H-NMR of PSB4 in DMSO-d_6.

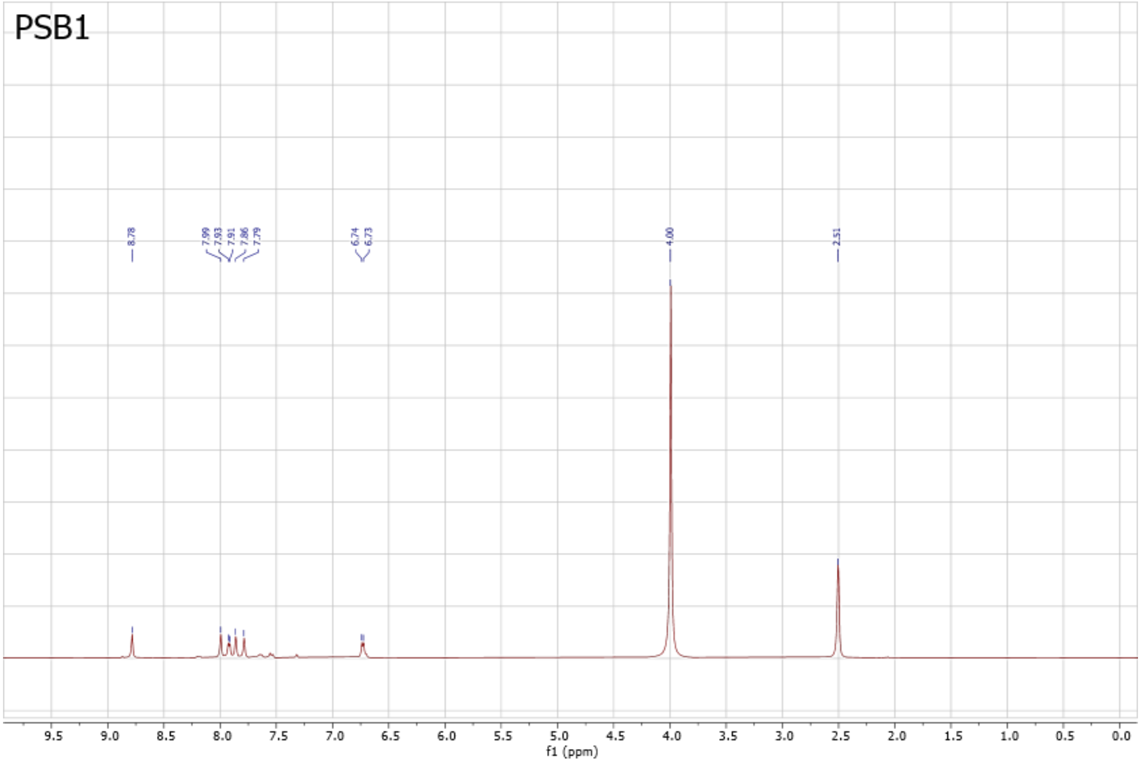

Figure S 22. D_2O exchange of PSB1 in DMSO-d_6.

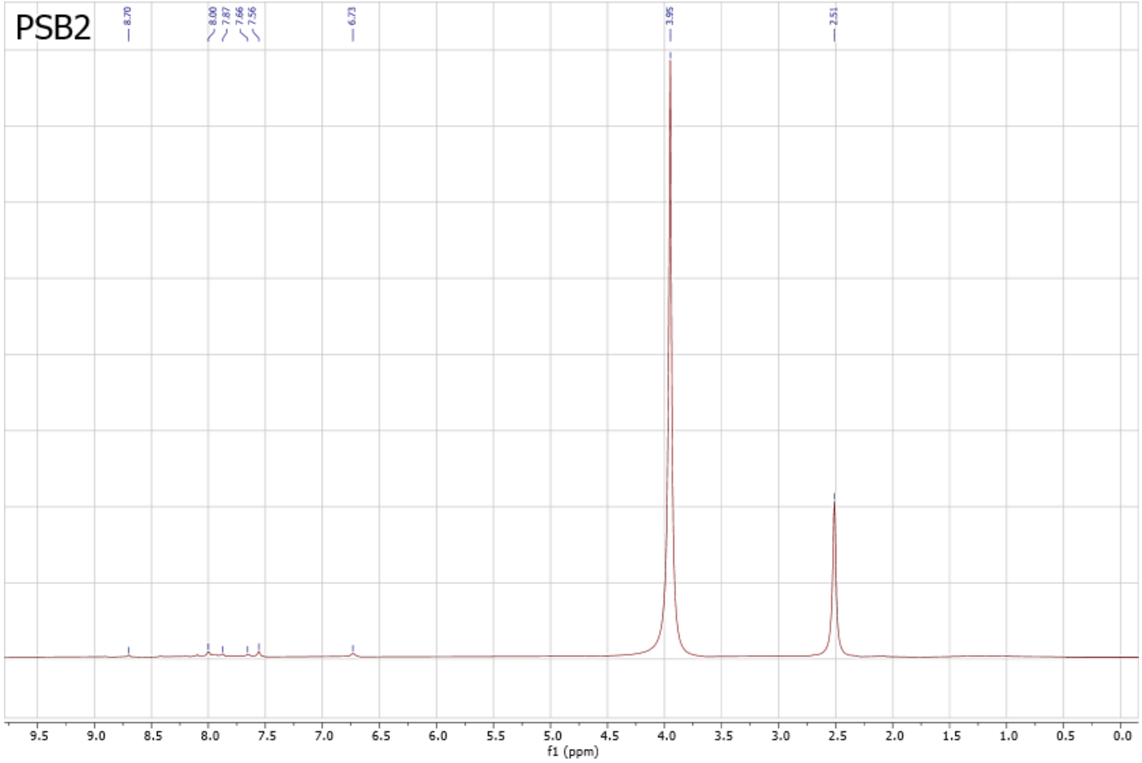

Figure S 23. D_2O exchange of PSB2 in DMSO-d_6.

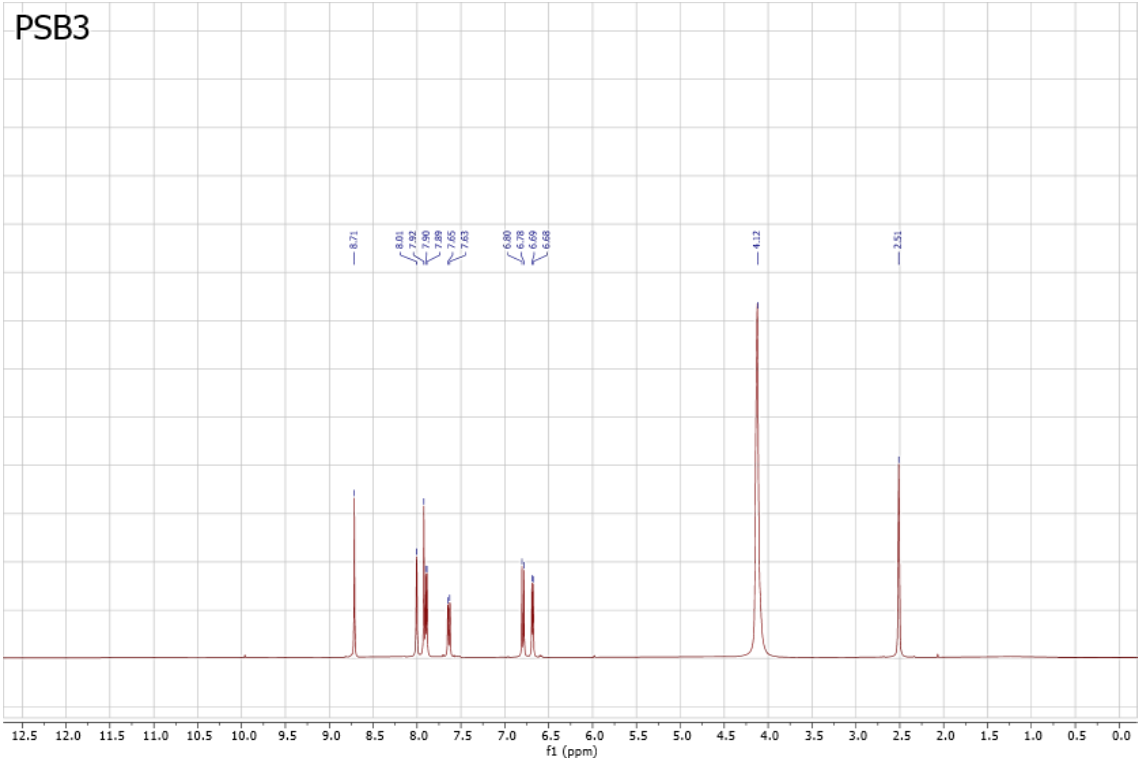

Figure S 24. D_2O exchange of PSB3 in DMSO-d_6.

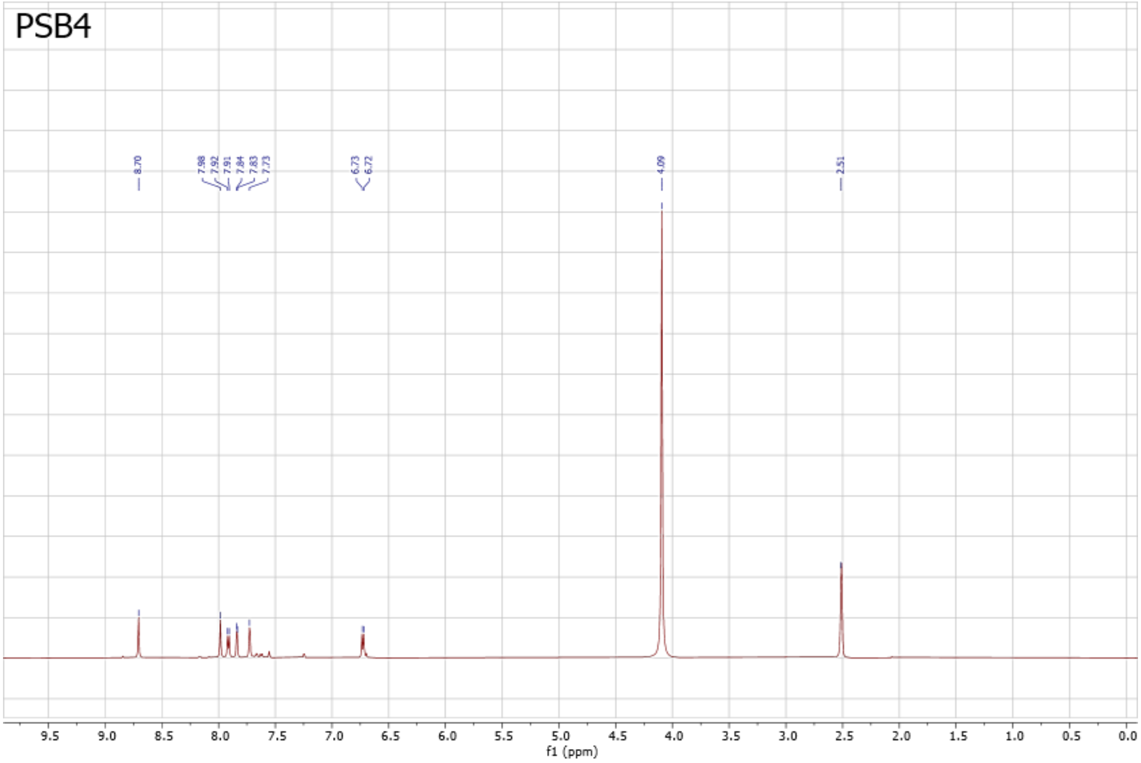

Figure S 25. D_2O exchange of PSB4 in DMSO-d_6.

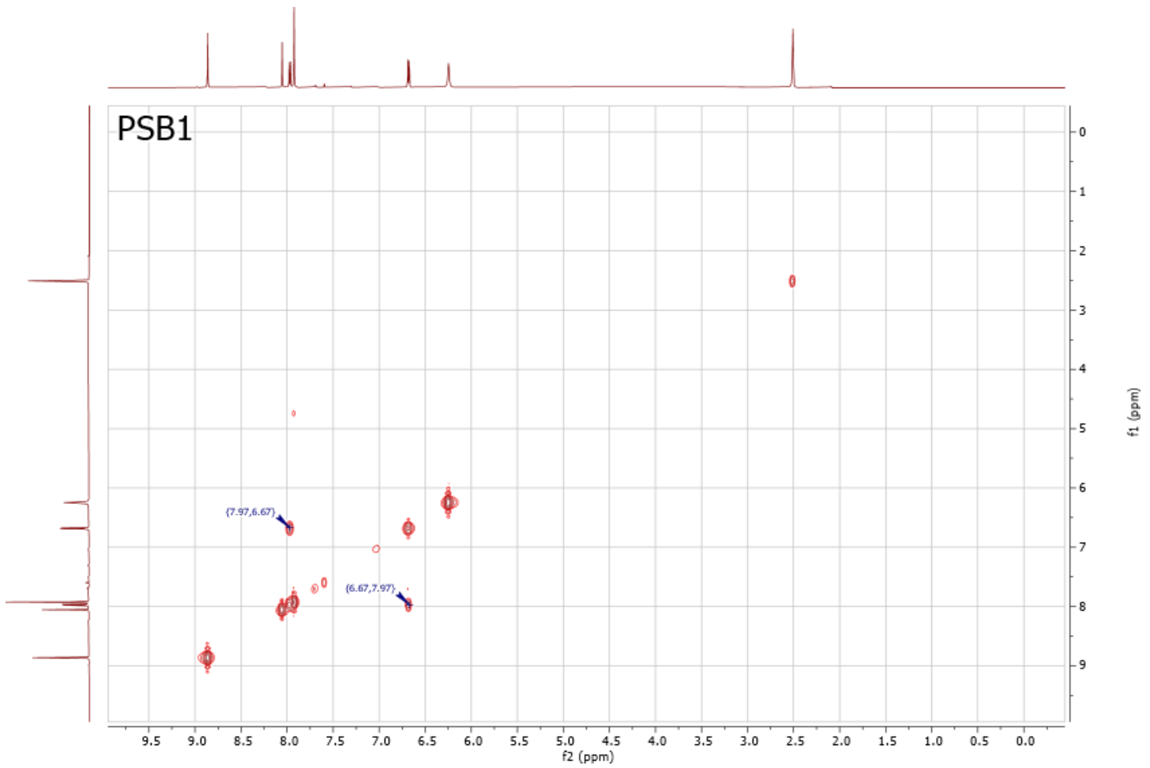

Figure S 26. HHCOSY of PSB1 in DMSO-d_6.

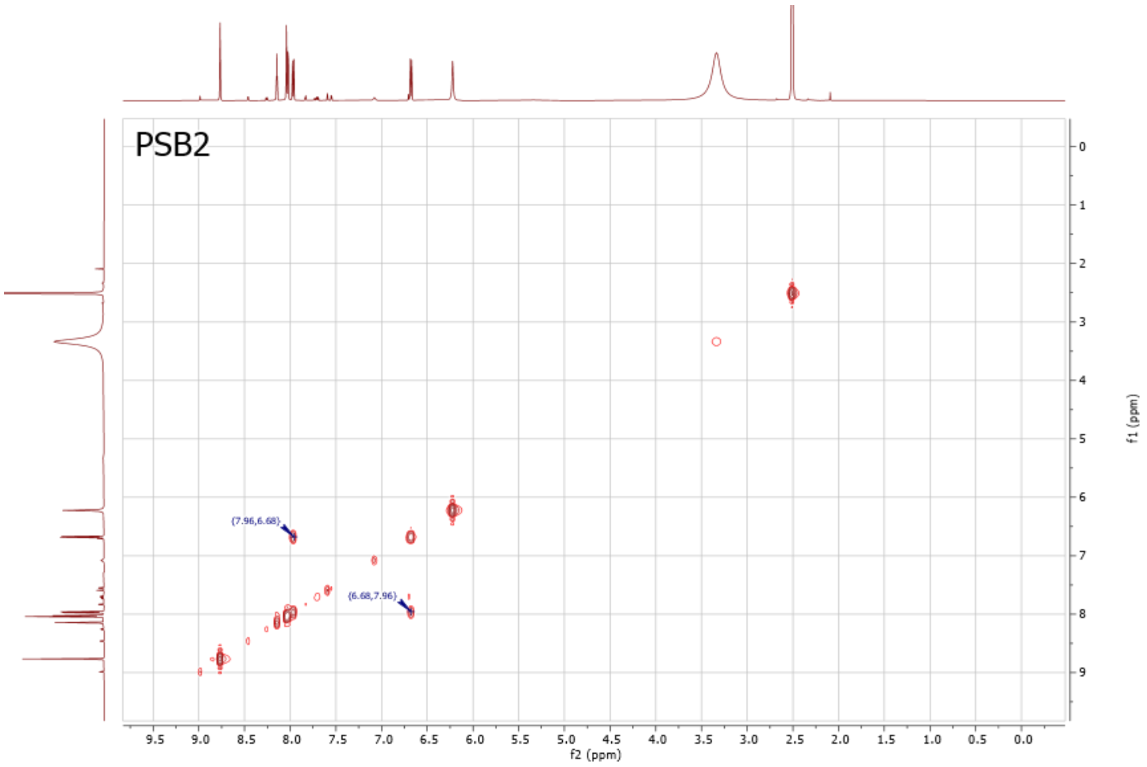

Figure S 27. HHCOSY of PSB2 in DMSO-d_6.

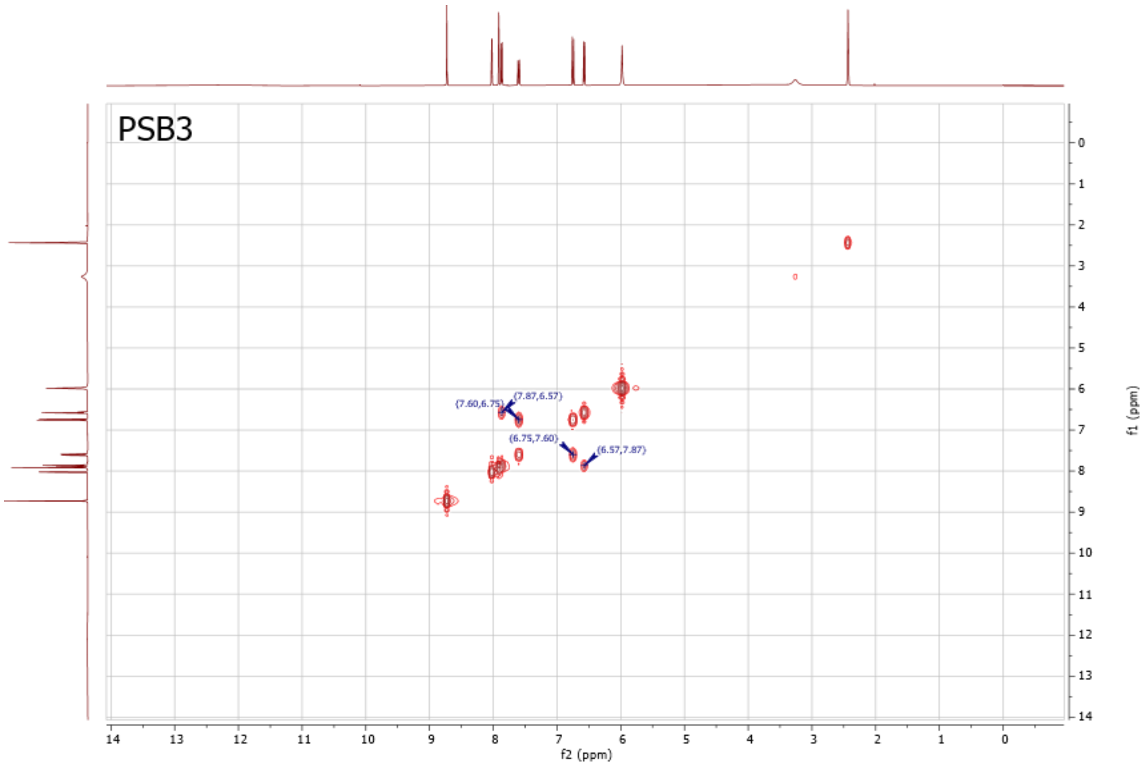

Figure S 28. HHCOSY of PSB3 in DMSO-d_6.

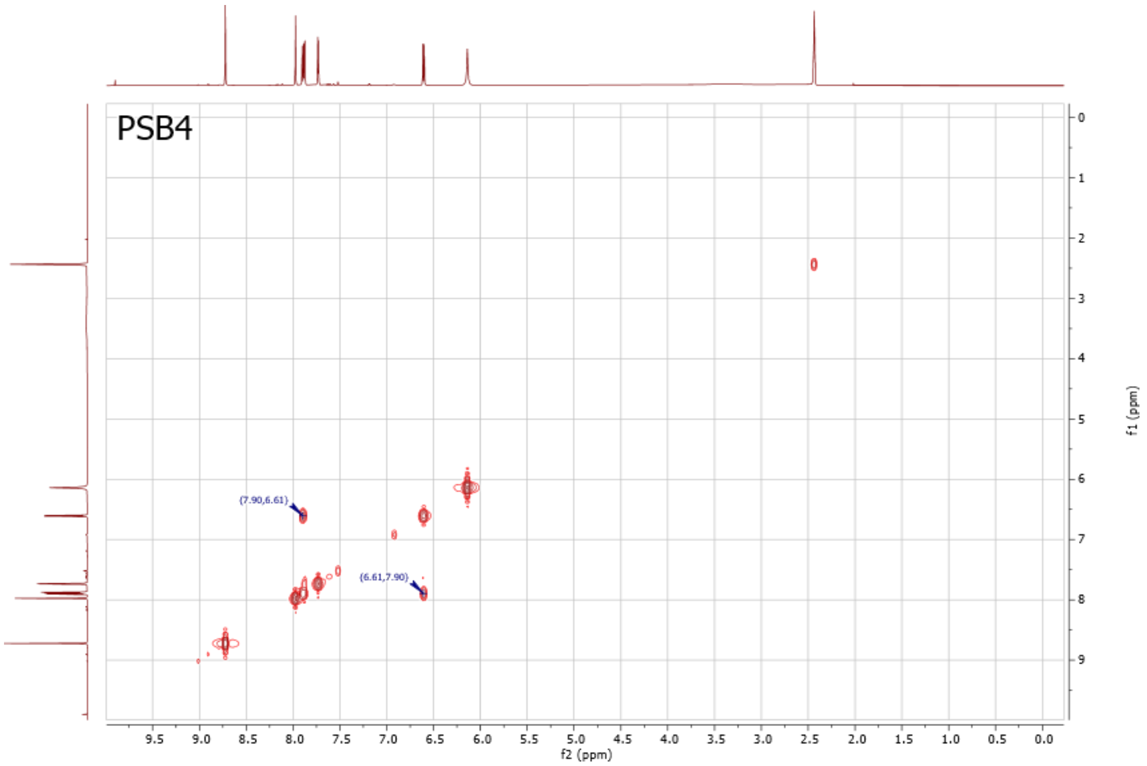

Figure S 29. HHCOSY of PSB4 in DMSO-d_6.

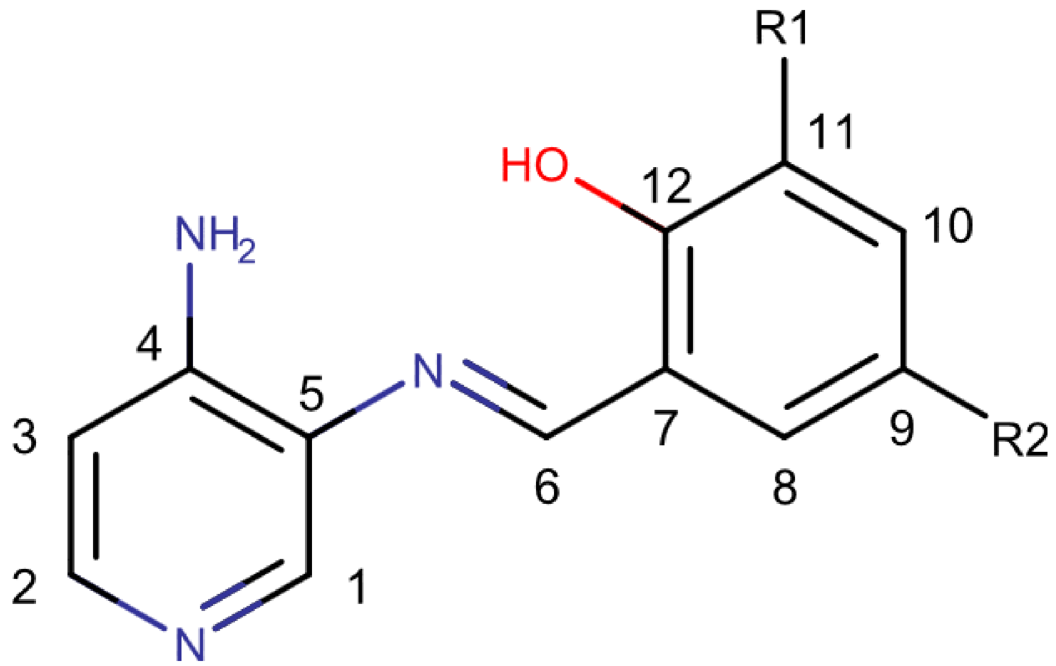

Figure S 30. Numbering atoms used in carbon assignments for PSBs.

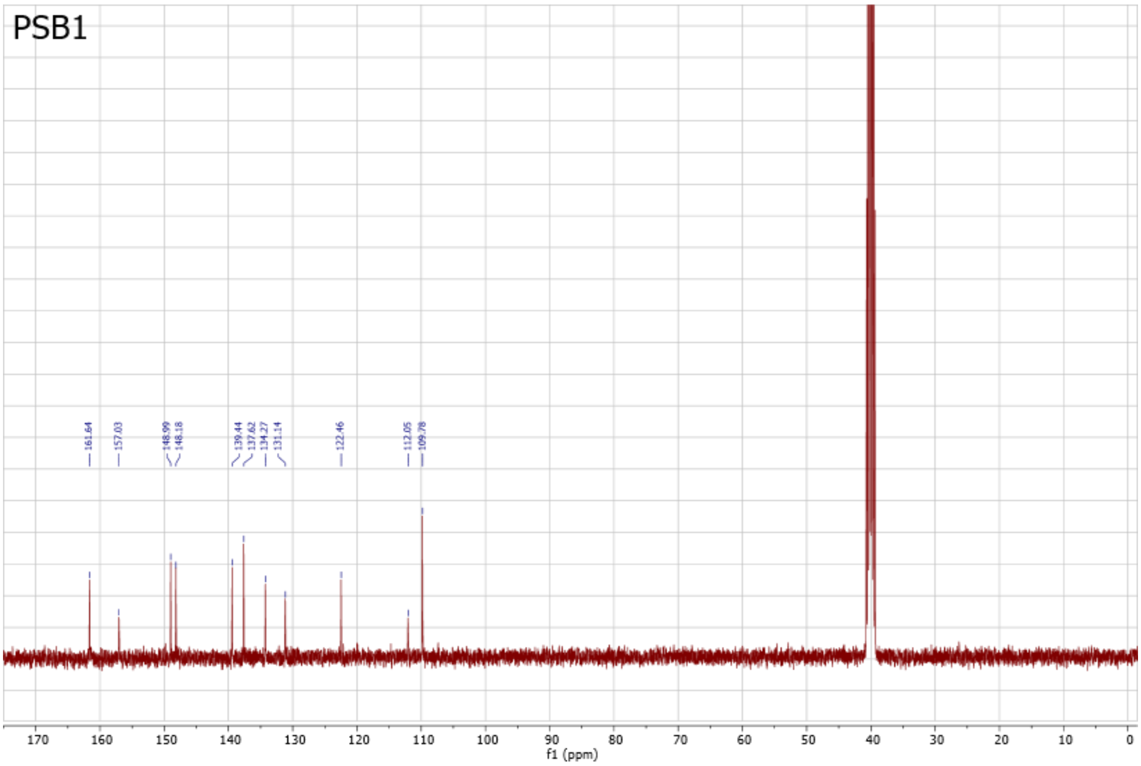

Figure S 31. ^{13}C-NMR of PSB1 in DMSO-d_6.

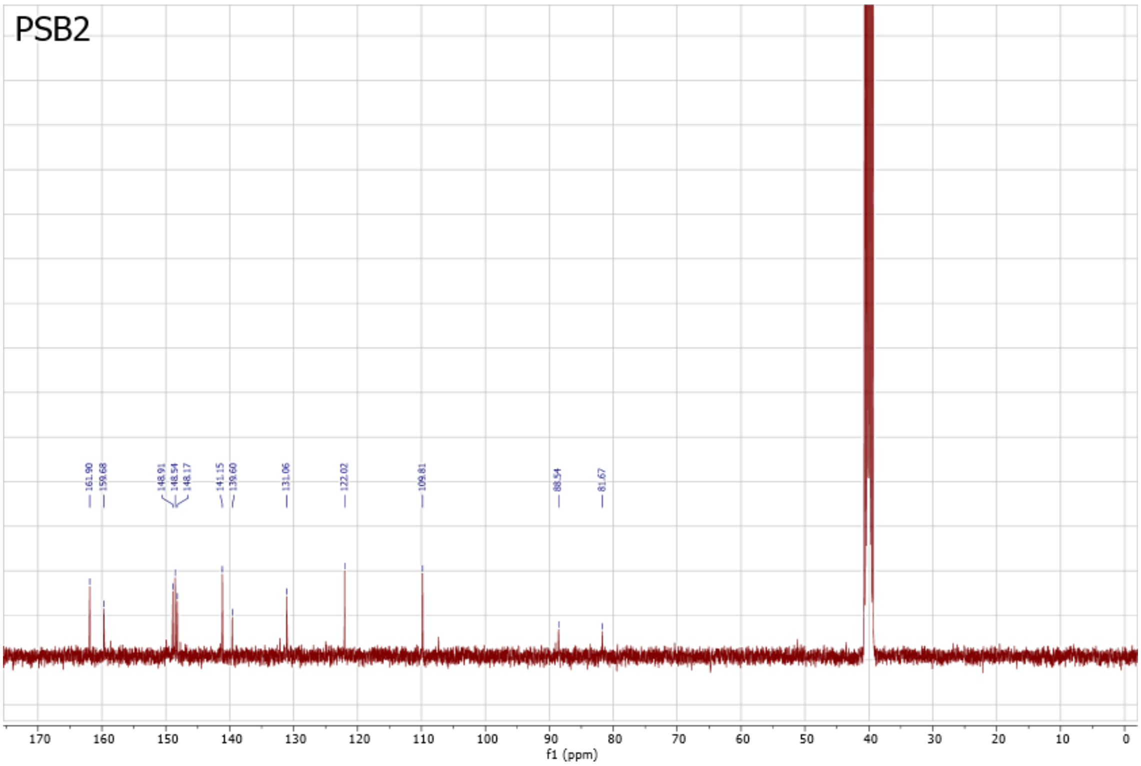

Figure S 32. ^{13}C-NMR of PSB2 in DMSO-d_6.

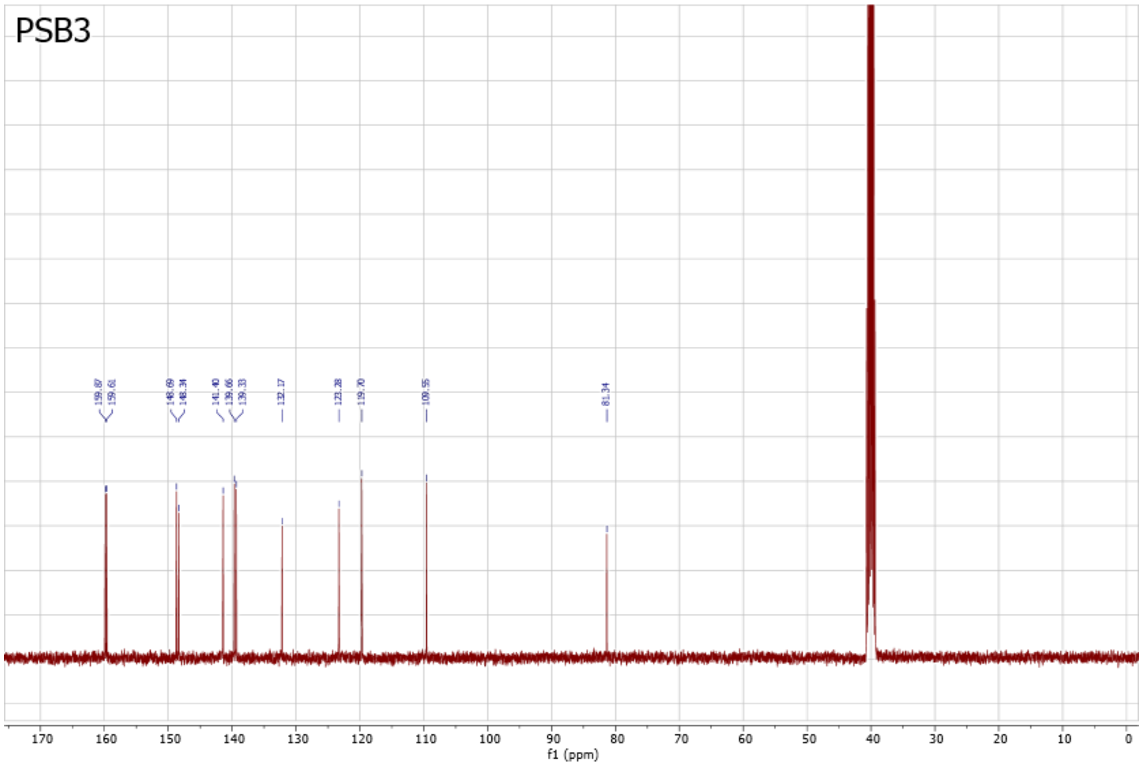

Figure S 33. ^{13}C-NMR of PSB3 in DMSO-d_6.

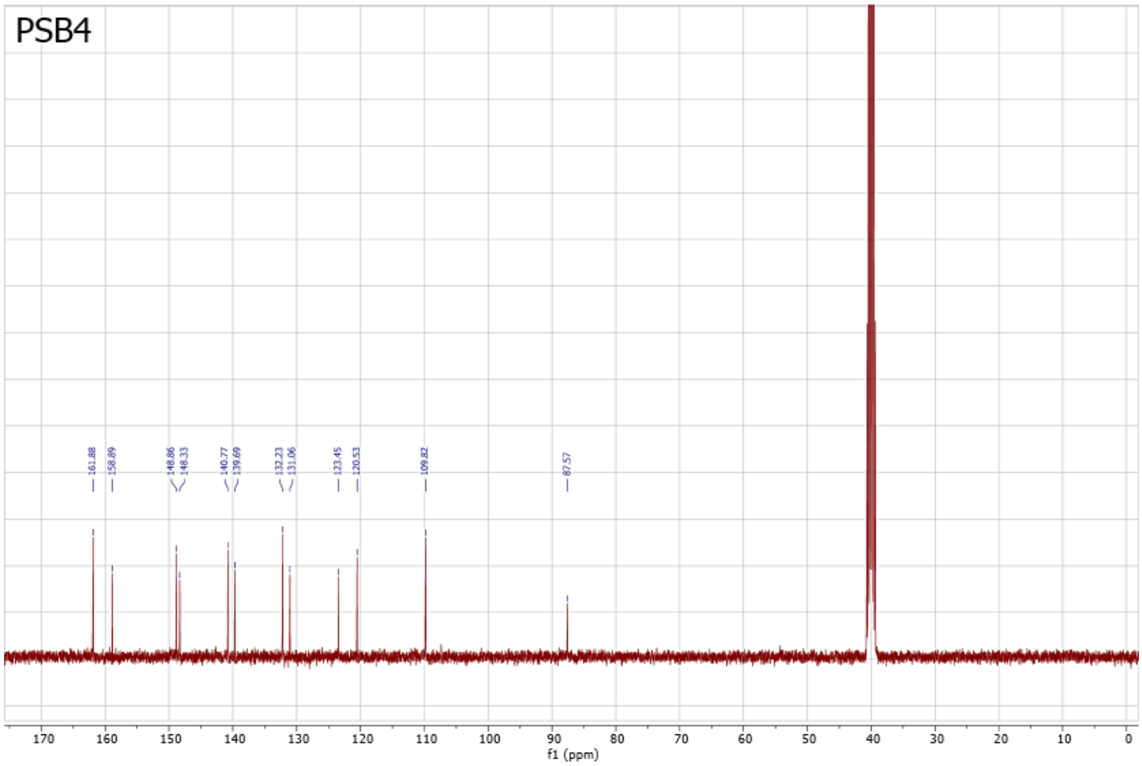

Figure S 34. ^{13}C-NMR of PSB4 in DMSO-d_6.

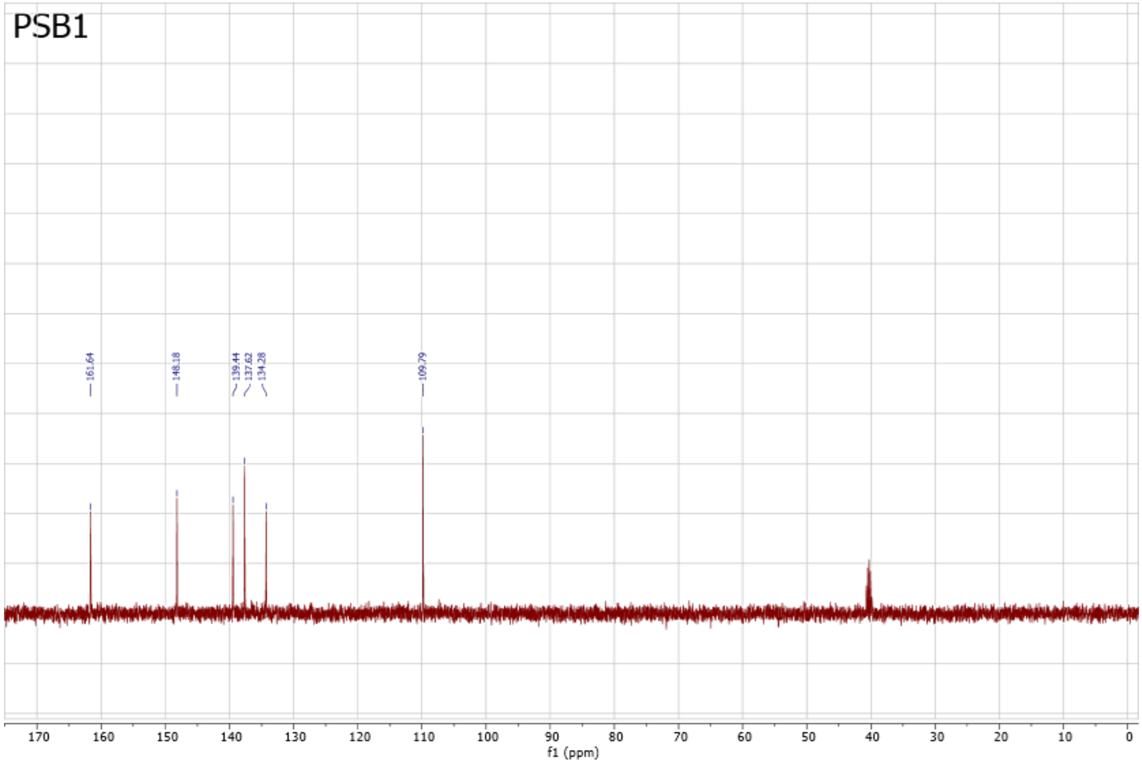

Figure S 35. DEPT 45 of PSB1 in DMSO-d_6.

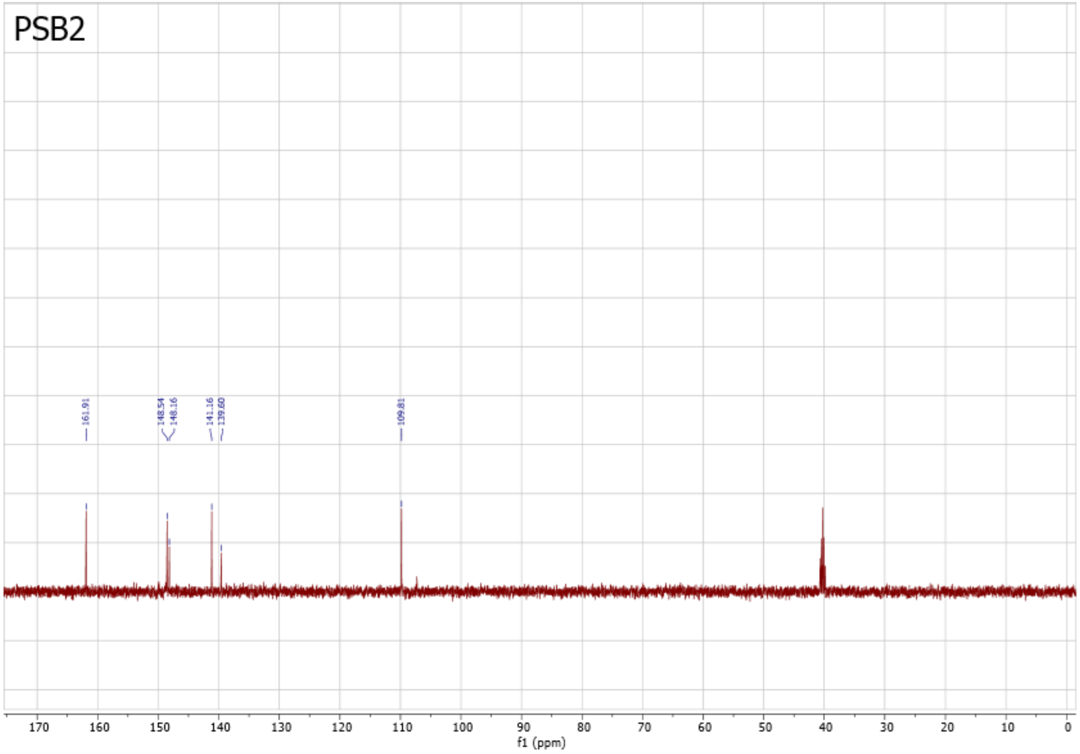

Figure S 36. DEPT 45 of PSB2 in DMSO-d_6.

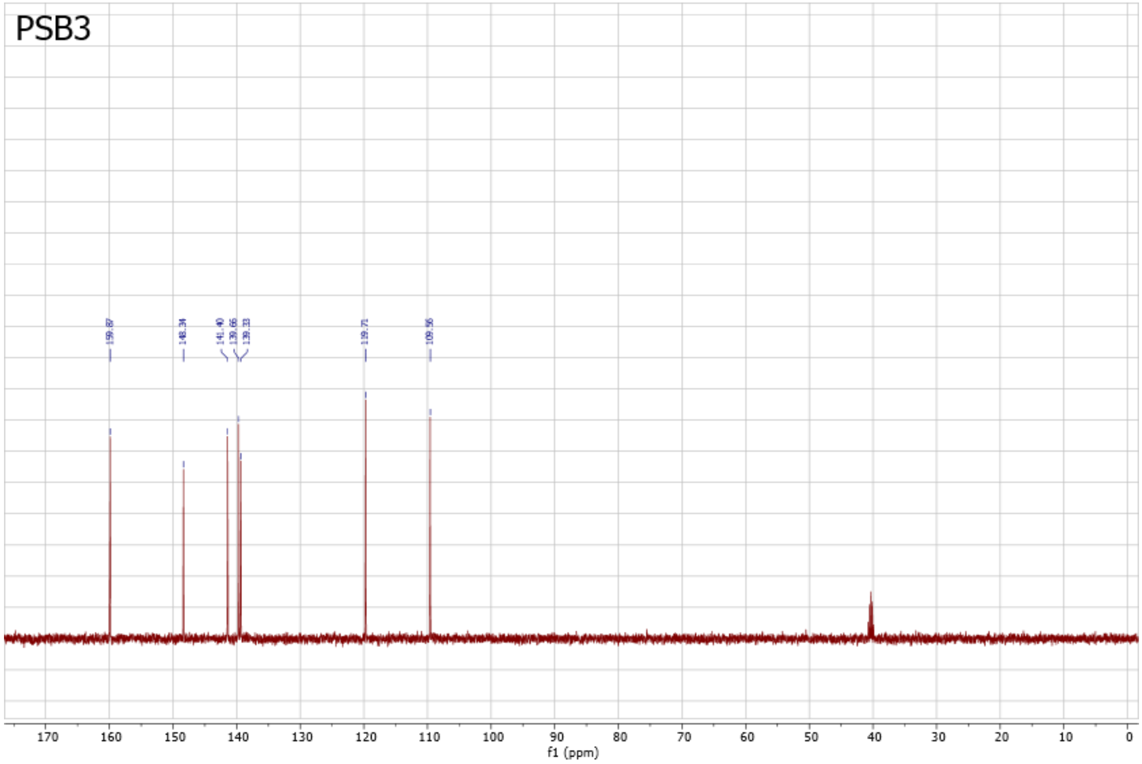

Figure S 37. DEPT 45 of PSB3 in DMSO-d_6.

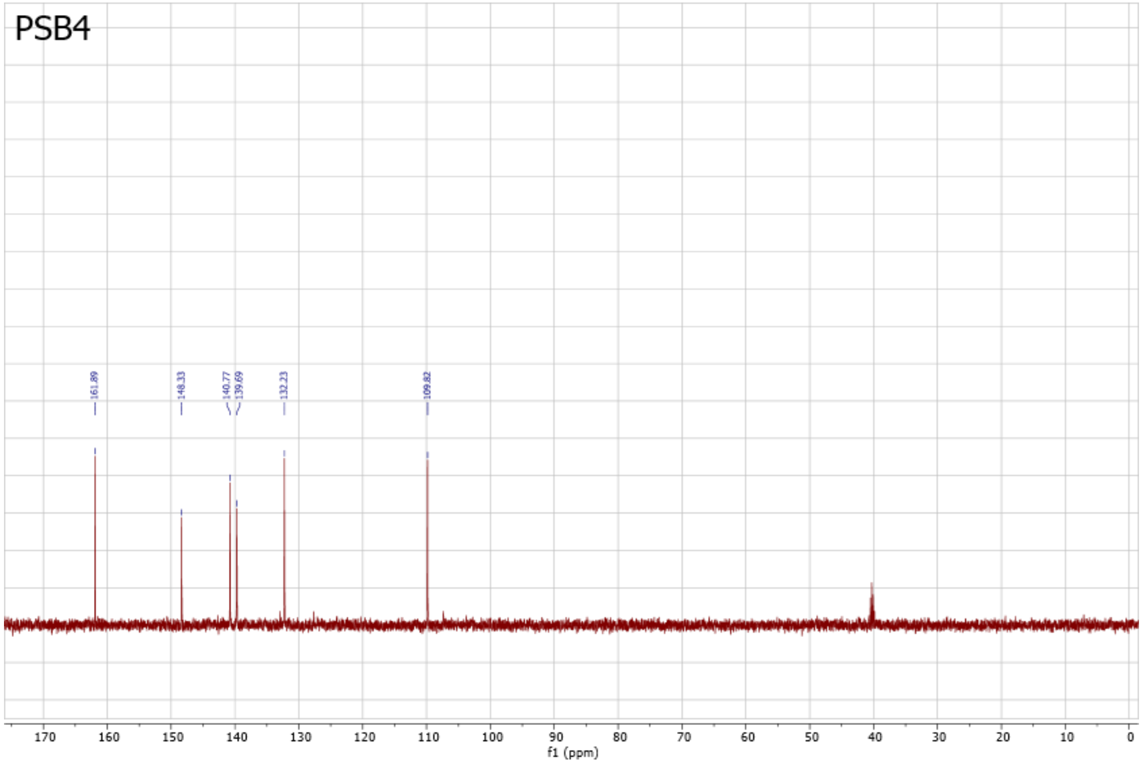

Figure S 38. DEPT 45 of PSB4 in DMSO-d_6.

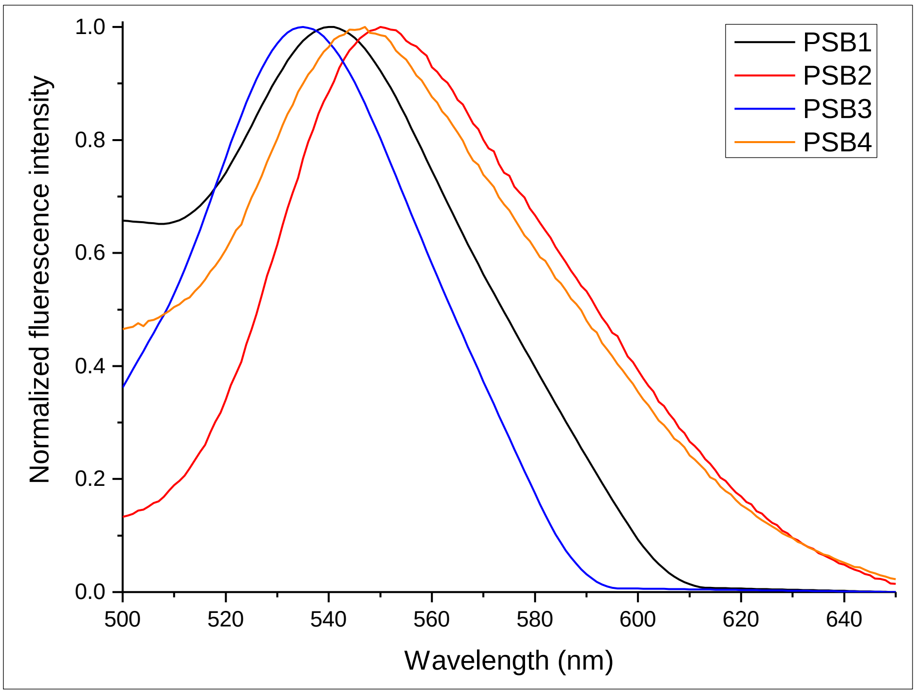

Figure S 39. Normalized emission spectra recorded in dichloromethane (DCM) of PSBs at room temperature.

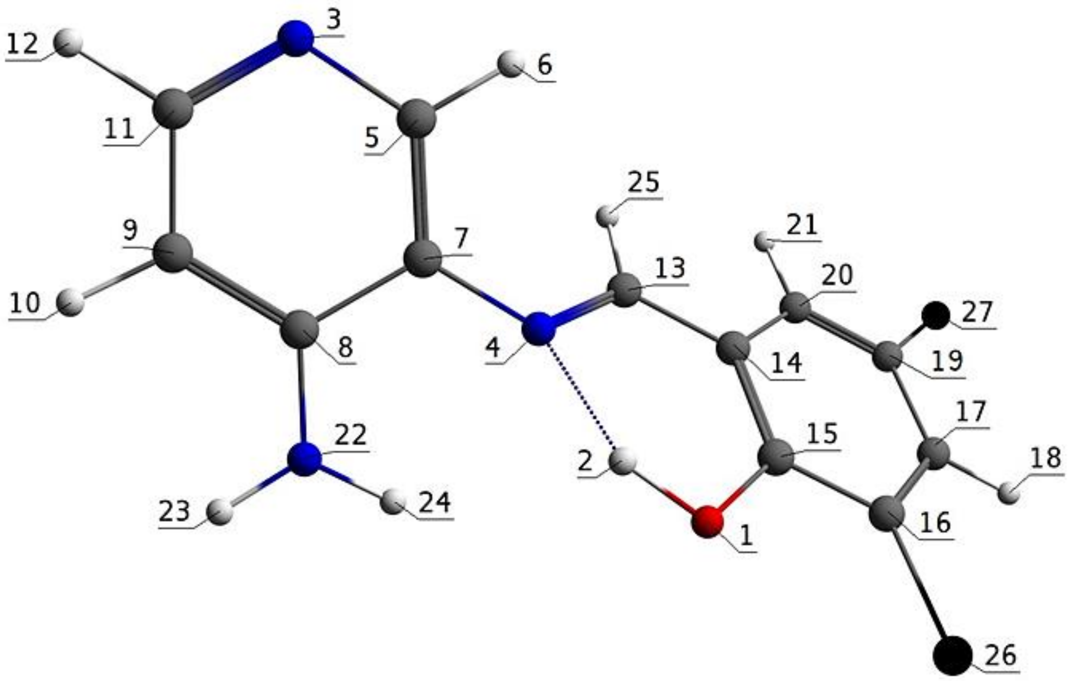

Figure S 40. Numbering atoms of PSBs with the different substitutions (black color) in the phenolic ring.
